# Efficient Preservation of Perishable Fruits by Erasable Metal‐Organic Frameworks

**DOI:** 10.1002/advs.202519222

**Published:** 2026-01-04

**Authors:** Liying Yang, Jia Kong, Haoran Bai, Xiaojie Wu, Douxin Xiao, Ming Du, Peng Yang, Alideertu Dong

**Affiliations:** ^1^ Engineering Research Center of Dairy Products Quality and Safety Control Technology College of Chemistry and Chemical Engineering Ministry of Education Inner Mongolia University Hohhot P. R. China; ^2^ Key Laboratory of Applied Surface and Colloid Chemistry School of Chemistry and Chemical Engineering Ministry of Education Shaanxi Normal University Xi'an P. R. China; ^3^ School of Dalian Ocean University Dalian P. R. China

**Keywords:** antimicrobial coating, ethylene scavenging, food preservation, metal‐organic frameworks, Pickering emulsion

## Abstract

With the worldwide crisis of food shortages, reducing postharvest losses in fruits and vegetables faces one of the greatest global challenges of contemporary food and agriculture. This study presented an erasable metal‐organic framework (MOF)‐based coating designed for dual functionality in combating foodborne pathogens and ethylene management. A MOF‐stabilized Pickering emulsion is developed to create a uniform and erasable protective coating on fruit surfaces through a simple dip‐coating process. The coating demonstrated an exceptional thermal/mechanical stability, a broad‐spectrum antimicrobial activity against phytopathogenic fungi (*Botrytis cinerea*, *Penicillium italicum*, and *Penicillium digitatum*) and bacteria (*Staphylococcus aureus* and *Escherichia coli*), and an effective ethylene scavenging capacity through the synergistic π‐complexation and hydrogen bonding interactions. As a protective layer, the coating reduced fruit weight loss by only 19.7% even after 10 days and captured ethylene production by 72.3%, markedly outperforming both commercial polyvinyl chloride films (20.8%) and the carboxymethyl chitosan‐based coating (45.6%). Metabolomics revealed the ability of the coating to downregulate ripening‐associated pathways, including protein digestion, amino acid metabolism, and glycolysis. Safety assessments confirmed the residue‐free washability of the coating, highlighting its sustainability for prolonging shelf life and mitigating postharvest losses.

## Graphical abstract

This study presents an erasable Pickering emulsion‐based coating stabilized by metal‐organic frameworks. This washable coating significantly extends fruit shelf life by scavenging ethylene and inhibiting foodborne pathogens, offering a sustainable solution for postharvest.

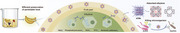

## 1 Introduction

Postharvest deterioration of fresh fruits and vegetables is a major cause of global food loss, particularly in long, complex supply chains where these products are vulnerable to microbial contamination, physiological senescence, and moisture loss [1, 2]. Consequently, food preservation coatings based on polysaccharides, proteins, and lipids have attracted increasing attention [3, 4, 5, 6]. Such biopolymer layers can form a conformal, dense barrier on the surfaces of fruits and vegetables, effectively slowing quality deterioration while avoiding additional plastic waste [7, 8]. In recent years, these protective coatings have further incorporated nanocomposite structures to enhance antimicrobial activity and gas‐barrier properties [9, 10]. Representative examples include curcumin‐protein coatings [11], tea polyphenol‐Zn^2^⁺ coatings [12], and resveratrol/chitosan coatings [13]. However, ethylene is also a key factor that promotes fruit ripening and accelerates decay. At present, most coating designs aim to optimize barrier properties or antimicrobial functions [4, 14, 15, 16]. Systems that can rapidly adsorb ethylene while simultaneously providing strong antimicrobial activity, remaining washable, and being safe for direct food contact are still extremely rare.

Active packaging strategies that modulate ethylene have shown great potential for extending fruit shelf life. Advanced polymer films or sachets can capture ethylene through chemical or physical adsorption [17, 18]. Porous metal‐organic frameworks (MOFs), with their high surface area, tunable porosity, and well‐defined binding sites, have emerged as promising sorbents for small‐molecule gases including ethylene [19, 20, 21]. Gaikwad et al. [22], Sultana et al. [8], and Liu et al. [23]. have recently reviewed MOF‐based active packaging systems in which MOF powders are dispersed in polymer matrices, fibrous mats, or sachets and act as ethylene or oxygen scavengers. Gammage and co‐workers further underscored the potential of MOFs in active food packaging, while at the same time highlighting concerns regarding direct ingestion and regulatory status [24]. To balance safety and performance [25], cyclodextrin‐based MOFs constructed from food‐derived ligands and alkali metal ions have been developed and employed as ethylene scavengers in direct contact with fruits such as kiwifruit [26, 27]. However, these MOF‐based systems are usually encapsulated in sachets, synthetic polymer films, or non‐washable composites [28, 29]. They fail to form water‐removable, food‐contact coatings that can be applied to fruit surfaces and subsequently removed.

Pickering emulsions (PE), in which solid particles irreversibly anchor at oil‐water (O/W) interfaces, offer a powerful platform for integrating hydrophobic antimicrobial agents into aqueous coating formulations [30]. In food systems, Pickering emulsions stabilized by biopolymers or proteins can control the release of essential oils and suppress microbial growth on fruit surfaces [31]. Yet coatings based on Pickering emulsions primarily target antimicrobial control, do not actively regulate ethylene, and are rarely designed to be fully washable or erasable after storage [7, 31].

**SCHEME 1:**
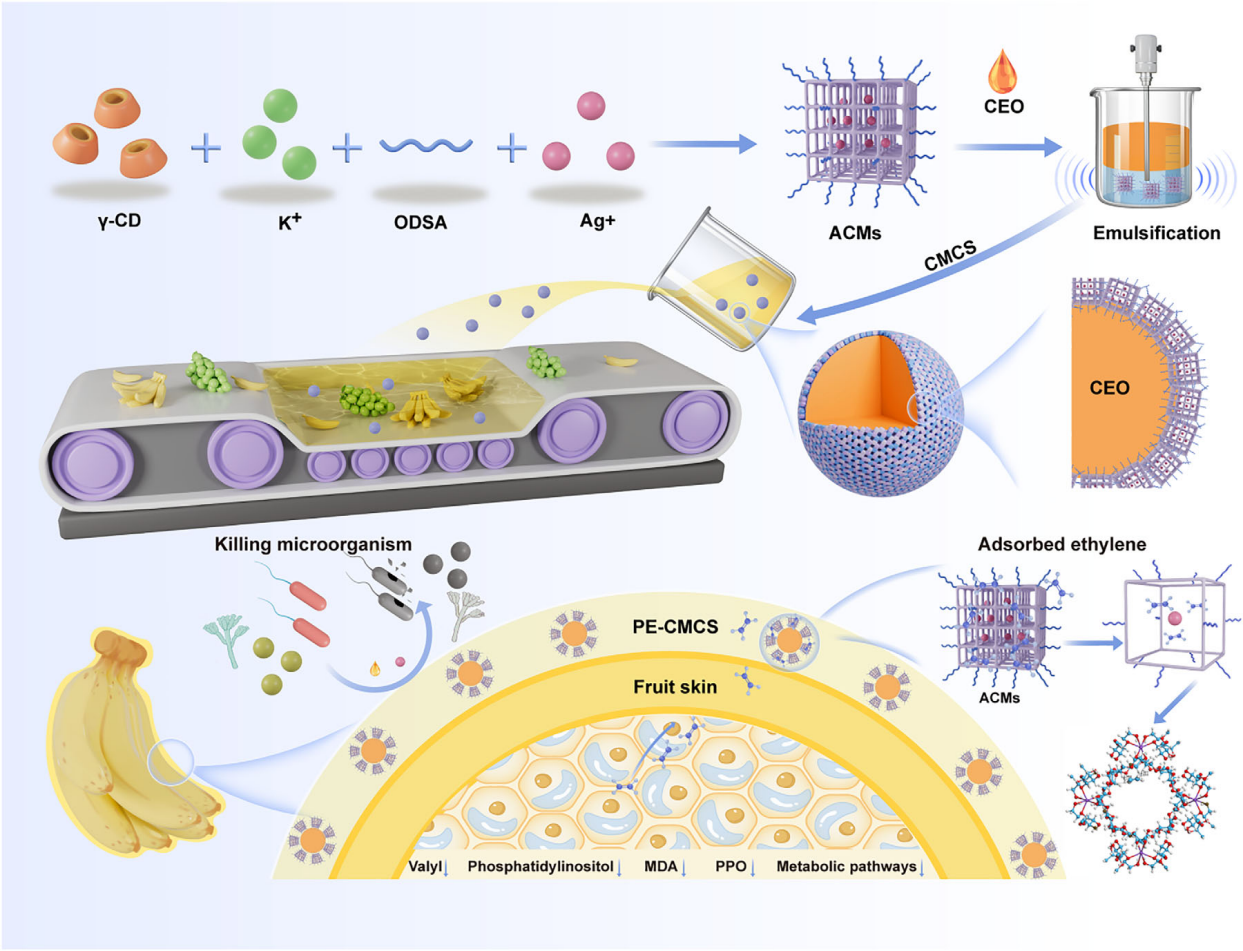
Illustration of the preparation of MOF‐based Pickering emulsions and their applications in the preservation of perishable fruits.

Here, we propose a MOF‐stabilized Pickering emulsion dispersed in a carboxymethyl chitosan (CMCS) matrix (PE‐CMCS) to create a washable coating that combines ethylene adsorption with broad‐spectrum antimicrobial activity, as schematically illustrated in Scheme 1. Engineered MOFs (denoted as ACMs) are used to stabilize clove essential oil (CEO) within the PE system, enabling sustained release to combat foodborne fungi and bacteria during storage. Notably, ACMs capture ethylene emitted from fruits via π‐complexation and hydrogen‐bonding interactions, effectively delaying fruit senescence. This strategy is compatible with most commonly used food preservatives and markedly increases the amount of preservative fixed on a wide range of fruits, achieving a loading over 2.9 times that of conventional commercial formulations. The PE‐CMCS coating can be completely removed by simple washing, thereby minimizing consumer exposure to metal‐containing components, and its safety is further clarified by comprehensive in vitro and in vivo assessments. We also systematically evaluate its water‐vapor and gas permeability relative to commercial polyvinyl chloride (PVC) and CMCS films, showing that the coating maintains sufficient oxygen and carbon dioxide transport to support fruit respiration while reducing weight loss. Collectively, these features establish a distinctive coating concept that integrates washability, ethylene management, antimicrobial activity, and balanced barrier properties to efficiently preserve perishable fruits and reduce postharvest losses.

## 2 Results and Discussion

### 2.1 Preparation and Characterization of PE

**FIGURE 1:**
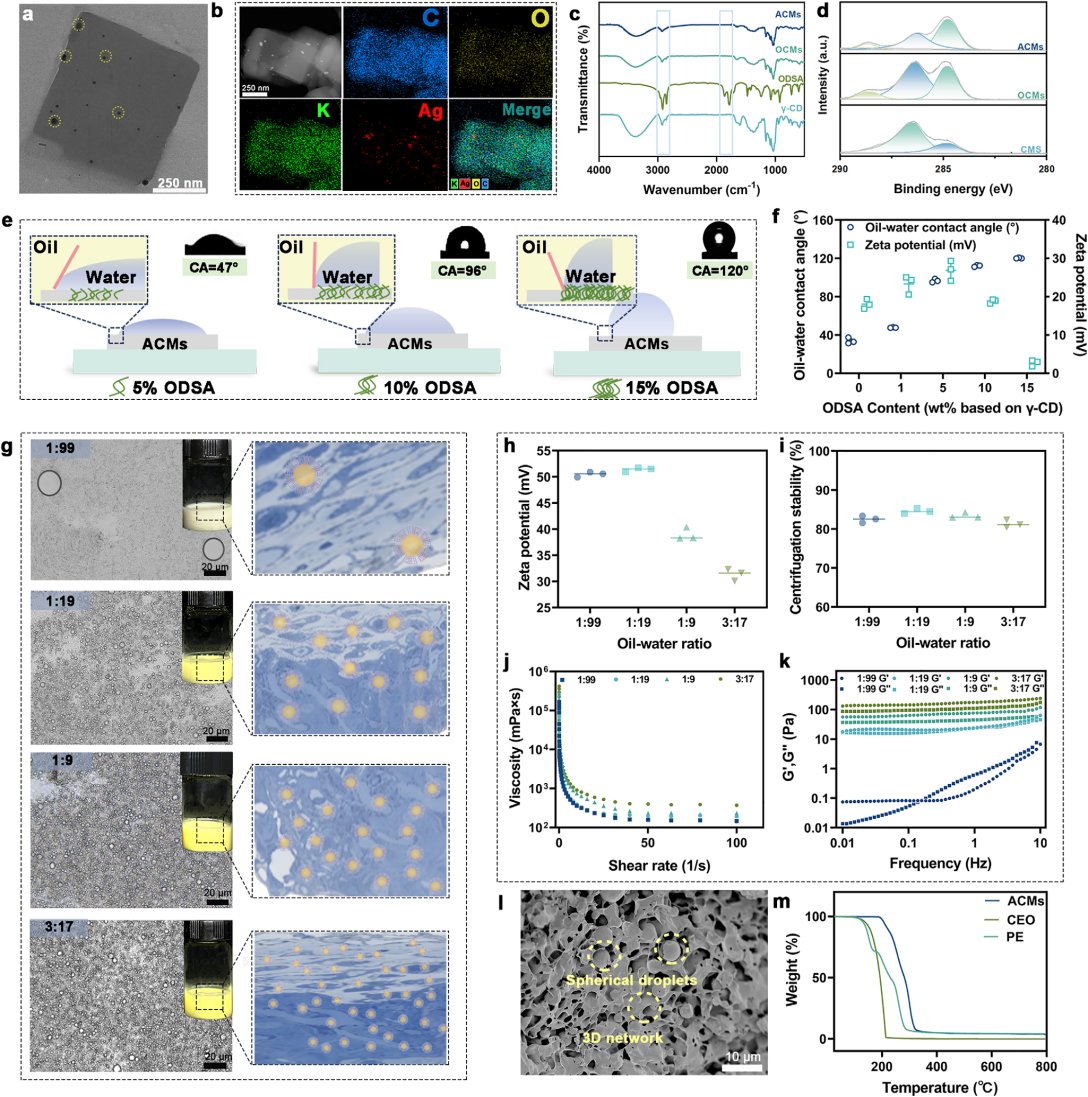
(a) TEM images and (b) element mapping of ACMs. (c) Infrared spectrum of CMs, ODSA, OCMs, and ACMs. (d) C 1s spectrum of XPS of CMs, OCMs, and ACMs. (e) Schematic illustration of the contact angle at the O/W interface of ACMs with different ODSA contents. (f) The contact angle and zeta potential of ACMs with different ODSA contents. (g) Microscopic images of PE at different oil/water volume ratios, respectively. (h) Zeta potential and (i) centrifugal stability constants, (j) viscosity, (k) storage modulus, and loss modulus at different oil/water volume ratios. (l) Cryo‐electron microscopy of PE under optimal conditions. (m) TGA curves of PE, CEO, and ACMs. Data are shown as mean ± SD (*n* = 3).

The synthesis process of the ACMs was illustrated in Figure S1. To enhance the hydrophobicity at the O/W interface, octadecenyl succinic anhydride (ODSA) was grafted onto the molecular structure of γ‐CD (γ‐cyclodextrin) to achieve ODSA‐CD. Subsequently, potassium ions (K^+^) were used as metal linkers to construct ODSA‐CD‐MOFs (OCMs). The generated OCMs have a stable structure and permanent porosity, and have been proven to possess functions such as gas storage, delivery, and degradation [32, 33, 34]. The ACMs were finally synthesized by utilizing the cavities of OCMs as nanoreactors for the reduction of silver ions. The morphology, structure changes, and evolution of MOFs during synthesis were monitored using SEM, TEM, elemental mapping, lattice analysis, FTIR spectroscopy, XPS, XRD, and electron diffraction (Figure 1a–d; Figures S2–S4). The ACMs displayed a tetrahedral morphology with a uniform diameter of 300 nm [35], while maintaining homogeneous nanoparticle (NP) dispersion within their framework (Figure 1a,b). Furthermore, based on the comparison of TGA data between OCMs and ACMs, an approximate loading of 5 wt.% NPs on ACMs was observed (Figure S3f).

We employed ACMs as stabilizers to prepare PE and subsequently investigated the factors influencing the stability of PE. Wettability was identified as a critical factor for PE stability [36], with optimal stability achieved when the contact angle approached 90° (Figure 1e). When the ODSA concentration was increased to 5 wt.%, the average water contact angle rose to 96.62 ± 1.87°, indicating that ACMs demonstrated a strong affinity for the oil phase and effectively stabilized at the O/W interface (Figure 1f). The concentration of ODSA also affected the zeta potential of ACMs, showing initially increasing then decreasing electronegativity due to carboxyl group ionization, which boosted negatively charged units and altered overall electronegativity [37]. Additionally, higher ODSA concentrations reduced the size of ACMs because ODSA's hydrophobic chains augmented intermolecular repulsion (Figure S5). The pH value significantly affected the stability of the PE system [38]. Acidic conditions destroyed the color of PE, turning it from yellow to white (Figure S6a). Additionally, the viscosity of the PE stabilized by ACMs rose with increasing pH, leading to the formation of more complex entanglements and micellar structures (Figure S6b,c) [39]. Zeta potential measurements exhibited a pH‐dependent trend (Figure S6d), with values decreasing from 57.63 ± 2.11 mV at pH 3 to 27.74 ± 0.73 mV at pH 9. The influence of pH on the centrifugal stability of PE was minimal, suggesting that ACM effectively maintained the stability of PE across a range of acidic and alkaline conditions (Figure S6e).

The stability of PE prepared with varying ACM concentrations was analyzed (Figure S7a). At lower concentrations (1.25 and 2.5 wt.%), instability and phase separation occurred due to inadequate encapsulation of the oil phase, leading to creaming. At 5 and 10 wt.%, ACMs effectively stabilized the O/W interface. Increased ACM concentration resulted in G' consistently exceeding G'', indicating a gel network protective layer at the O/W interface (Figure S7b, Supporting Information). Higher ACM concentrations also enhanced solution viscosity due to increased solubility and electrostatic repulsion (Figure S7c) [40]. Zeta potential values exceeded 30 mV across all concentrations from 1.25 to 10 wt.%, confirming emulsion stability (Figure S7d) [41]. Centrifugal testing showed the lowest stability at 2.5 wt.%, with 5 wt.% selected as the optimal condition for stabilizing PE (Figure S7e).

PE samples with varying oil/water ratios were then analyzed. The results showed a slight decrease in zeta potential as CEO content increased, while centrifugal stability remained unaffected by oil/water ratio adjustments (Figure 1h,i). Figure 1g,j demonstrated that increasing the oil phase percentage enhanced CEO droplet density and emulsion viscosity. However, oil/water ratios of 1:9 to 3:17 led to excessive viscosity, complicating emulsion coating for food preservation. Except for the 1:99 ratio, where lower CEO content resulted in G′ < G″, all other solutions exhibited a stable elastic gel‐like structure characterized by G' > G″ (Figure 1k). Optimal stabilization conditions for PE were identified as 5 wt.% ACM concentration, pH 7, and an oil/water ratio of 1:19. Cryo‐electron microscopy (Figure 1l) revealed a dense and uniform droplet distribution, with ACMs forming a network‐like structure that inhibited droplet coalescence. Confocal laser scanning microscopy (CLSM) confirmed the O/W interface structure, showing red oil droplets surrounded by green ACM aqueous solution (Figure S8). TGA analysis (Figure 1m) indicated that adding CEO slightly reduced PE thermal stability.

### 2.2 Antimicrobial Properties and Mechanism of PE

**FIGURE 2:**
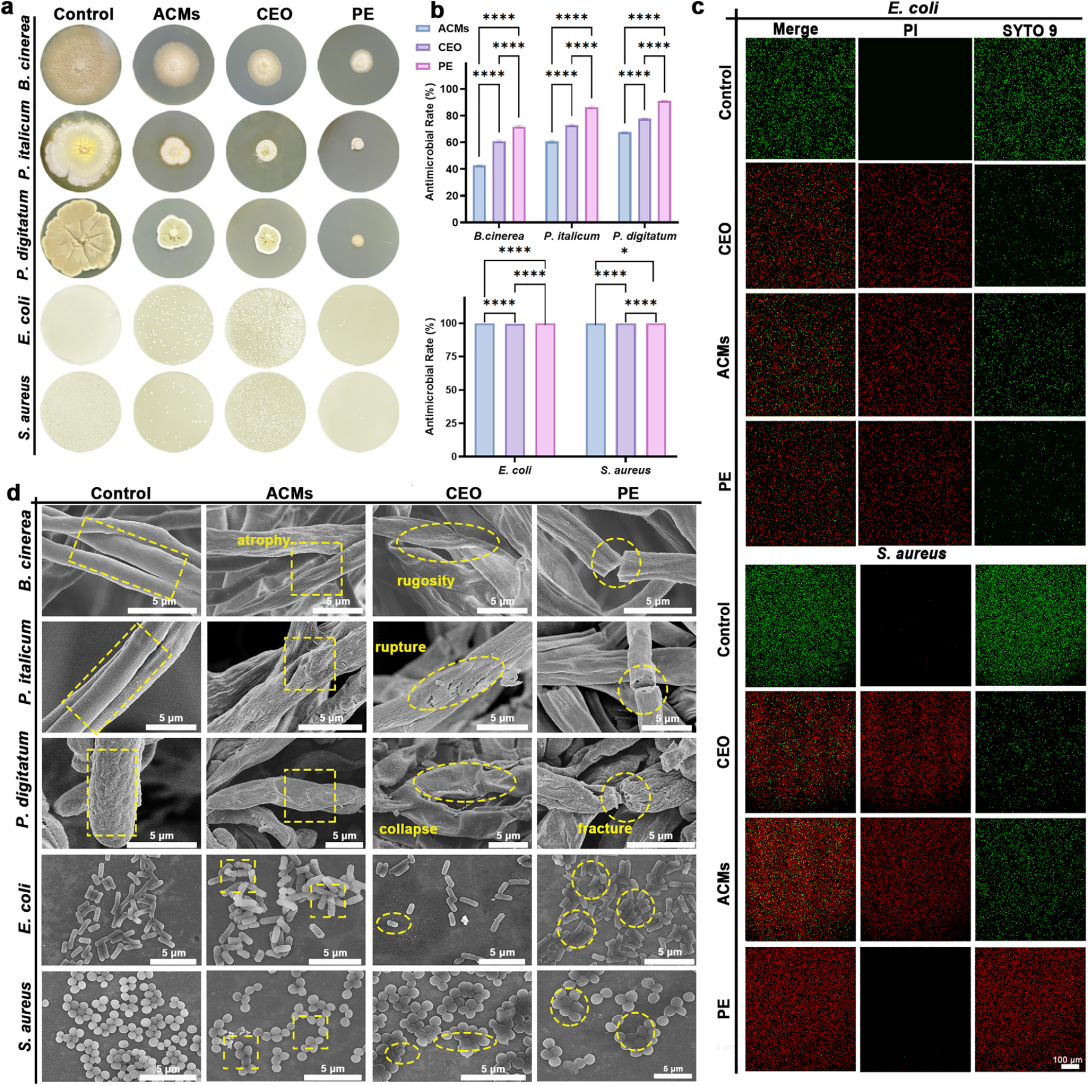
(a) The antimicrobial effect and (b) inhibition rate of CEO, ACMs, and PE against *B. cinerea*, *P. italicum*, *P. digitatum*, *E. coli*, and *S. aureus*. (c) CLSM images of *E. coli* and *S. aureus* stained with PI and SYTO9 after treatment with CEO, ACMs, and PE, respectively. (d) SEM images of *B. cinerea*, *P. italicum*, *P. digitatum*, *E. coli*, and *S. aureus* treated with CEO, ACMs, and PE, respectively. Data are shown as mean ± SD (*n* = 3), and *p* values were obtained by one‐way ANOVA followed by Duncan's post‐hoc test. Statistical significance is set as *p < 0.05, **p < 0.01, ***p < 0.001, ****p < 0.0001; ns, not statistically significant.

For the three major postharvest fungal pathogens (*Botrytis cinerea*, *Penicillium italicum*, and *Penicillium digitatum*) [42], the PE showed the highest inhibition rates, reaching approximately 80%–90%. The CEO followed with inhibition levels of 60%–75% (Figure 2a,b). All intergroup differences were highly significant (****, *p* < 0.0001). These results indicated that incorporating CEO into the Pickering emulsion enhanced its antifungal performance. In addition, all three treatments achieved uniformly high antibacterial activity (>98%) against *E. coli* and *S. aureus* [43]. Nevertheless, significant differences were still observed, with the PE showing the highest inhibition level (>99.99%), followed by ACMs and CEO. The strong antibacterial effect of the PE was mainly attributed to eugenol in CEO, which disrupted membrane fluidity and interacted with essential cellular components [44]. The incorporation of CEO and ACMs into the PE did not diminish antibacterial efficacy, suggesting that the active components were effectively released to exert their bactericidal action.

We investigated the evolution in bacterial cell membrane permeability using CLSM [45]. Bacteria treated with ACMs and CEO displayed red fluorescence, which was more intense in samples treated with PE, suggesting that PE induced bacterial death by compromising the integrity of the cell membrane (Figure 2c). Microstructural analysis further corroborated this finding (Figure 2d). Under CEO treatment, the bacterial cell membrane exhibited only partial contraction (indicated by ellipses), whereas ACMs treatment caused marked membrane disruption with evident leakage of intracellular contents (squares). Following PE treatment, bacterial cells exhibited substantial structural damage and marked morphological alterations (circles). Analogous effects were observed in fungal mycelium. Under normal conditions, the mycelium displayed a regular morphology characterized by straight, robust, and uniformly sized filaments with smooth surfaces (rectangles). However, upon ACM treatment, the mycelial filaments underwent pronounced contraction (squares). Exposure to CEO resulted in severe wrinkling and dehydration of the hyphae (ellipses). Moreover, PE exposure induced cracking and fragmentation of the hyphae (circles).

### 2.3 Ethylene Adsorption and Mechanism

Brunauer‐Emmett‐Teller (BET) analysis and computational simulations were performed to assess the ethylene‐capturing capacity of ACMs. Based on adsorption‐desorption curve analysis, the specific surface area of γ‐CD was calculated to be 0.6749 m^2^/g with a pore size of 31.4064 nm (Figure S9). When γ‐CD was coordinated with metal cations to form ACMs, its specific surface area increased significantly to 76.9803 m^2^/g, while the pore size was 9.08 nm (Figure S10). We also evaluated the ethylene adsorption capacity of ACMs and OCMs. As shown in Figure S11, the adsorption isotherms clearly demonstrated that ACMs exhibited significantly higher ethylene adsorption at both low and high relative pressures (P/P_0_), with a maximum adsorption capacity approaching 2 cm^3^/g (STP). In contrast, OCMs showed much lower adsorption (about 1 cm^3^/g), indicating that the incorporation of Ag enhanced ethylene capture capacity.

**FIGURE 3:**
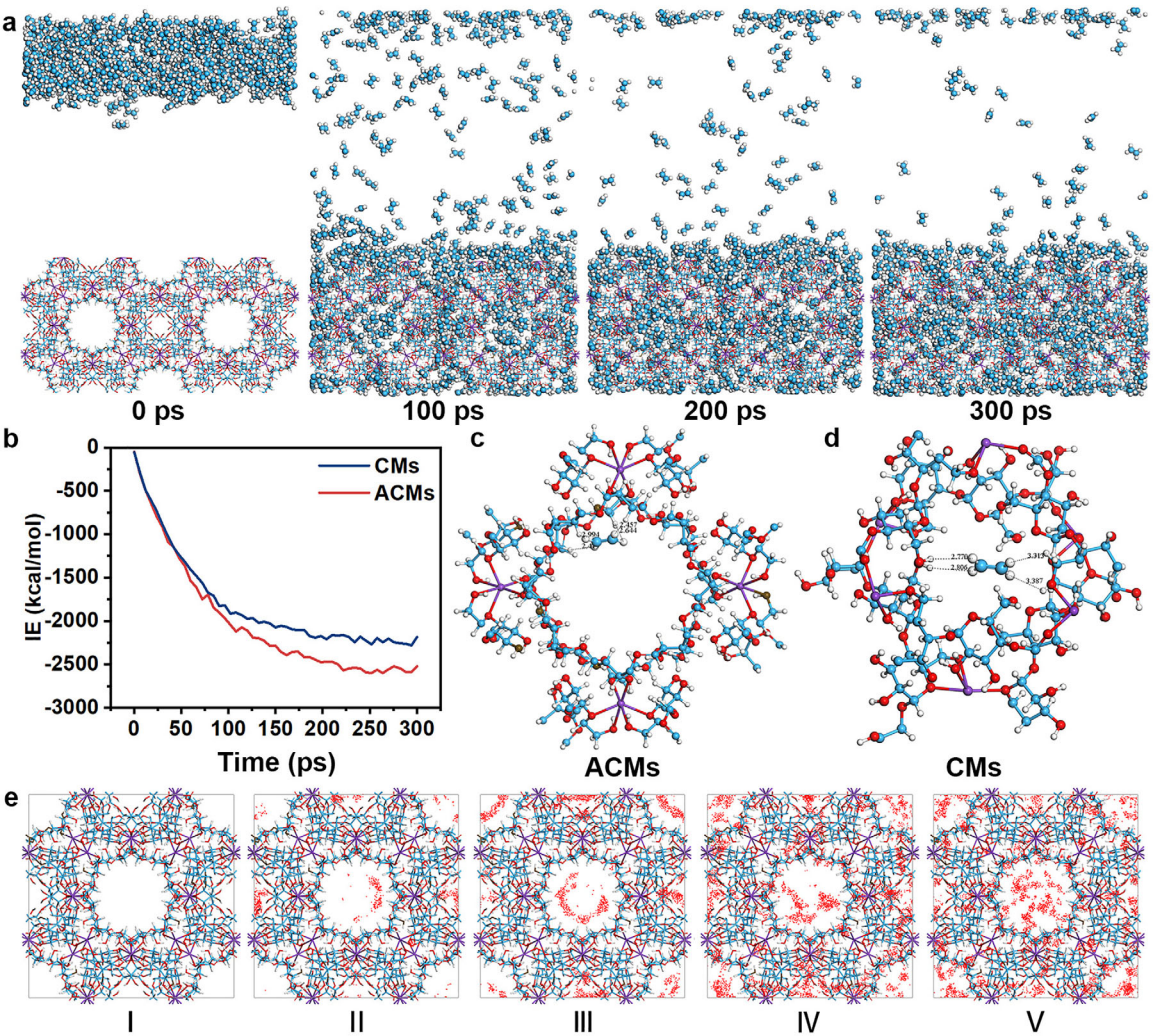
(a) Adsorption of ethylene on ACMs simulated by the Metropolis method over 300 ps. (b) The interaction energies between MOFs (CMs or ACMs) and ethylene molecules. GCMC simulation‐derived adsorption of ethylene on (c) ACMs and (d) CMs. (e) Ethylene distribution density within ACMs at 298 K, with densities increasing sequentially from I to V.

The adsorption behavior of ethylene molecules on MOFs and their mechanisms was studied by theoretical calculation methods. The adsorption of ethylene molecules on CMs and ACMs was simulated using the Sorption module in Materials Studio (MS) 2020. Figure 3a and Figure S12 showed the complete adsorption process of ethylene molecules entering the microporous channels, showing their spontaneous diffusion into the ACMs framework, followed by molecular entrapment. Computational revealed adsorption potential energies of −2521.887 kcal/mol for ACMs and −2188.073 kcal/mol for CMs, indicating a spontaneous adsorption process with superior adsorption capacity in ACMs compared to CMs (Figure 3b). Hydrogen atoms in both frameworks exhibited the closest proximity to ethylene molecules. Specifically, the calculated distances between hydrogen atoms on ethylene and the nearest hydrogen atoms in ACMs were 2.419, 2.457, 2.644, and 2.944 Å (Figure 3c), whereas the corresponding distances in CMs were 2.770, 2.806, 3.313, and 3.387 Å (Figure 3d). This indicated stronger interfacial interactions between ethylene and ACMs. The density distributions of ethylene in ACMs and CMs are illustrated in Figure 3e and Figure S13, respectively. At low concentrations of ethylene, the peripheral cavities of ACMs initially adsorbed ethylene molecules. As the ethylene concentration progressively increased (as evidenced by the red dots indicating the density distribution of ethylene), ethylene molecules subsequently began to fill the large central cavity of the ACMs.

### 2.4 Biocompatibility Evaluation of PE‐CMCS Coating

**FIGURE 4:**
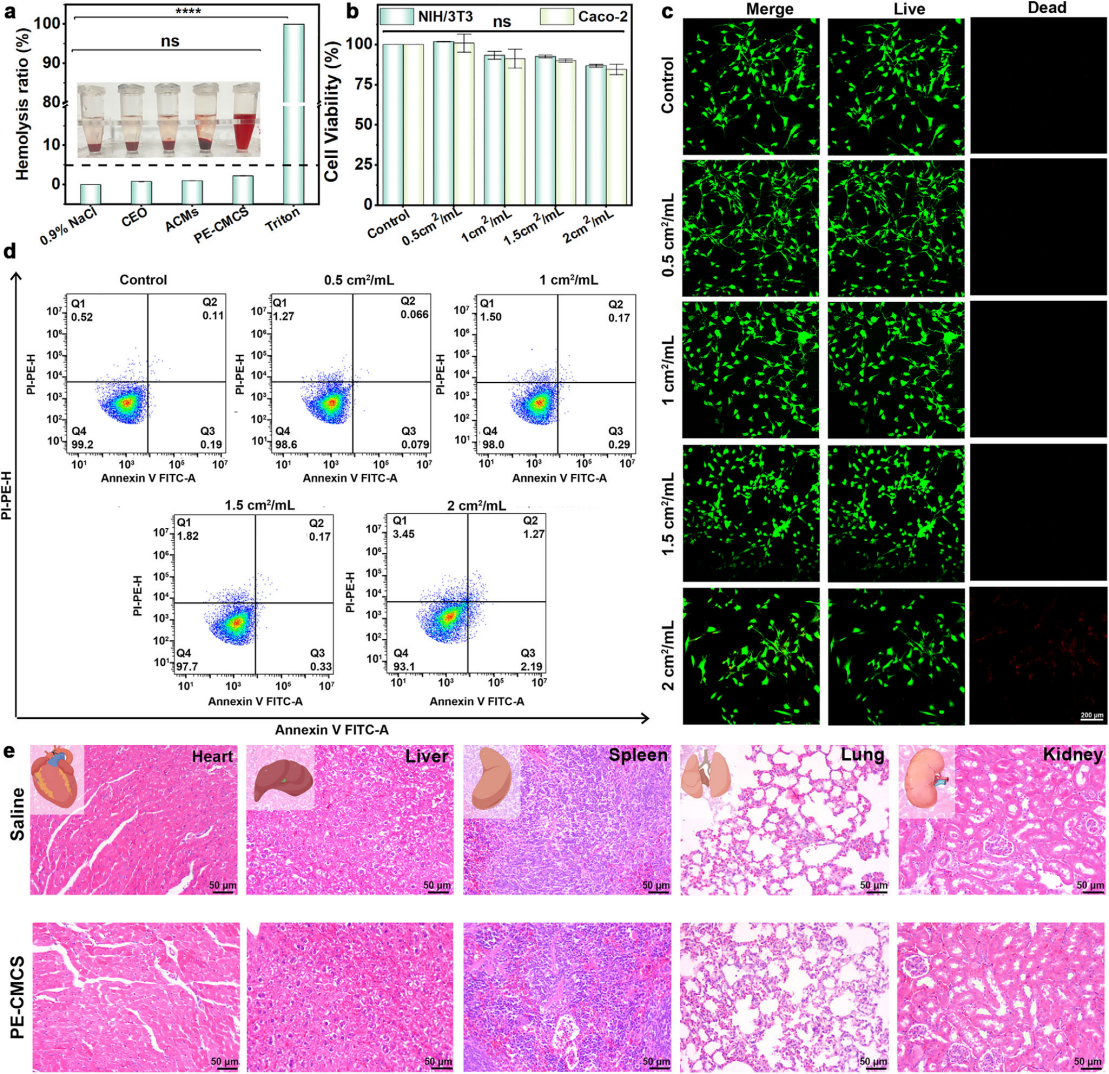
(a) Hemolysis rate and digital photos of CEO, ACMs, and PE‐CMCS. (b) Effect of different areas of PE‐CMCS film leachate on cell viability. (c) Live/dead staining after culturing NIH 3T3 cells in a cell culture medium with different areas of PE‐CMCS film leachate for 24 h. (d) Apoptosis rates of NIH 3T3 cells treated within a cell culture medium with different areas of PE‐CMCS film leachate for 24 h. (e) H&E staining of the heart, liver, spleen, lung, and kidney of the mice after treatment with saline and PE‐CMCS. Data are shown as mean ± SD (*n* = 3), and *p* values were obtained by one‐way ANOVA followed by Duncan's post‐hoc test. Statistical significance is set as *p < 0.05, **p < 0.01, ***p < 0.001, ****p < 0.0001; ns, not statistically significant.

The safe application of the PE‐CMCS coating in fruit preservation was verified through a systematic evaluation of its toxicological safety and food‐grade suitability. The hemolysis rates of the supernatants from PE‐CMCS and its individual components were all below the 5% threshold, demonstrating that these materials exhibit no hemolytic activity (Figure 4a) [45]. Cell viability assays using mouse embryonic fibroblasts (NIH 3T3) and human colon adenocarcinoma cells (Caco‐2) further confirmed low cytotoxicity. Even at high concentrations (2 cm^2^/mL), cell viability remained above 80% (Figure 4b). Live/dead staining revealed predominantly viable cells across all concentrations (Figure 4c). Furthermore flow‐cytometric analysis demonstrated that >90% of NIH 3T3 cells remained in the viable quadrant, with no appreciable increase in early or late apoptosis after PE‐CMCS treatment (Figure 4d). These data indicate that potential leachates from the coating did not induce overt membrane damage or apoptosis in representative mammalian cell lines.

Systemic biosafety was corroborated in vivo. Following intragastric administration of PE‐CMCS, the mice exhibited no signs of weight loss or behavioral abnormalities (Figure S14). And H&E staining of major organs (heart, liver, spleen, lung, kidney) revealed no histopathological lesions compared with saline controls (Figure 4e). Routine hematology parameters, including white blood cells, granulocytes, and lymphocytes, remained within normal physiological ranges (Figure S15). Moreover, the MOF employed in PE‐CMCS is constructed from food‐grade γ‐CD and K⁺ [46, 47, 48]. Among the other main components, clove essential oil is recognized as a Generally Recognized As Safe (GRAS) flavoring substance by the U.S. Food and Drug Administration and has been evaluated by EFSA as an authorized food flavor [49, 50]. Additionally, carboxymethyl chitosan is a biocompatible polysaccharide that has been widely applied in food‐contact and biomedical materials [51, 52, 53, 54, 55, 56]. These findings strongly support that the coating is biocompatible and non‐toxic under the tested conditions, making it suitable for direct contact with fresh fruits in practical food preservation applications.

### 2.5 Spreadability, Washability, and Freshness‐Preserving Properties of Coating

**FIGURE 5:**
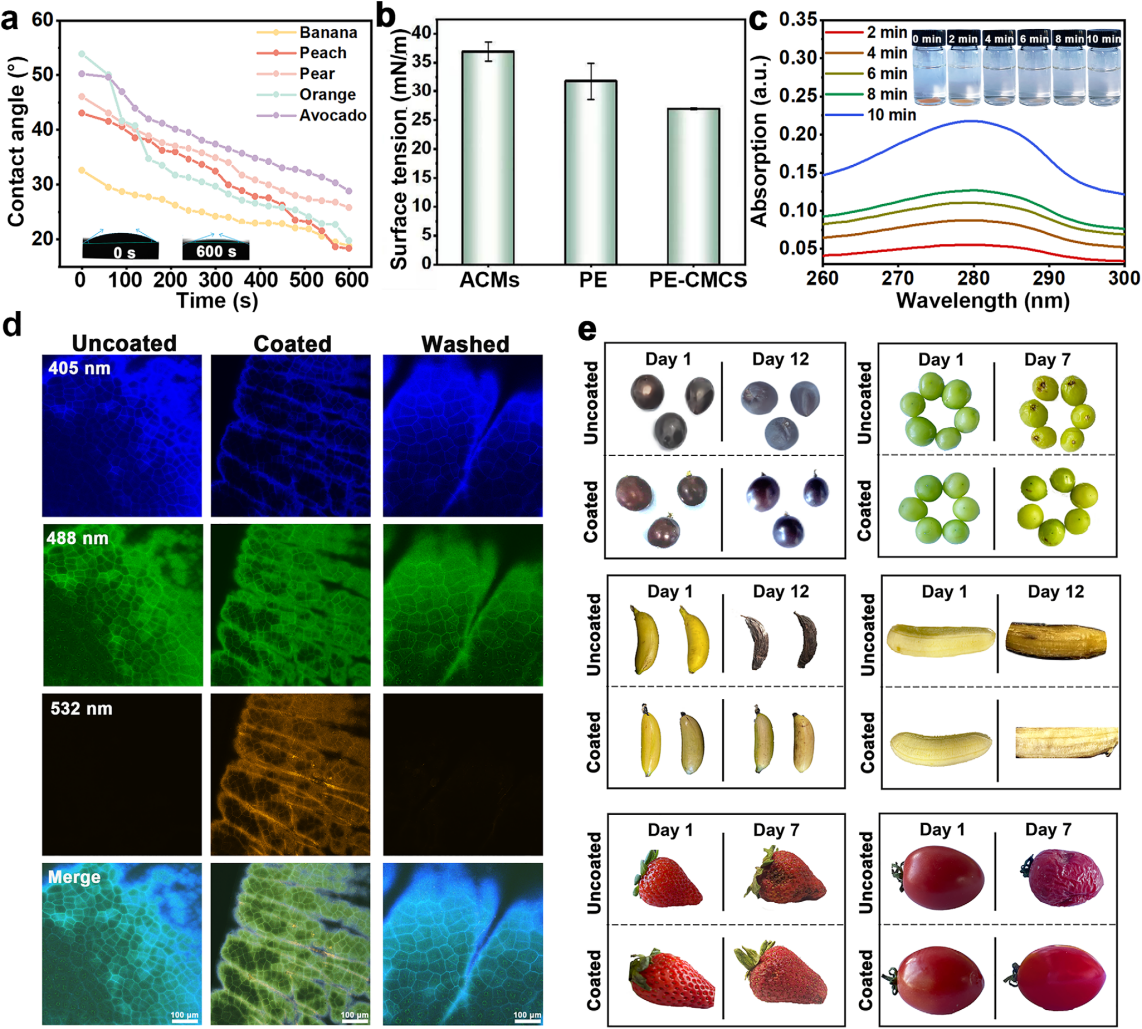
(a) Contact angles of PE‐CMCS solution on banana, peach, pear, orange, and avocado peel surfaces at different times. (b) Surface tension of ACMs, PE, and PE‐CMCS in air. (c) UV absorption spectra during PE‐CMCS film dissolution. (d) CLSM images of uncoated, coated, and post‐washing coated Shine Muscat grapes at different excitation wavelengths. (e) Digital images of Kyoho grapes, Shine Muscat grapes, bananas, strawberries, and cherry tomatoes after being coated with PE‐CMCS. Data are shown as mean ± SD (*n* = 3).

Figure S16 depicts the dip‐coating process employed for the preparation of washable fruit coatings, as well as its effectiveness in preservation. Understanding the wetting behavior and hydrophilicity of the coating solution on the surfaces of fruits with different degrees of roughness is crucial for determining how the solution forms a uniform surface coating. As shown in Figure 5a, the contact angle on the surface of a banana decreased from an initial 32.61 ± 0.46° to 18.77 ± 0.13° within 10 min after wetting. Similarly, affected by the roughness, the initial contact angles of peaches, pears, oranges, and avocados were 43.05 ± 2.69°, 46.03 ± 0.60°, 53.87 ± 1.05°, and 50.23 ± 0.21°, respectively, but all decreased by at least 42% within 10 min. In contrast, the contact angle of pure water decreased by only 18% within 10 min (Figure S17). This indicates that the coating has a strong affinity and wettability for diffusing onto the fruit surfaces [11]. It is worth noting that bananas exhibit the smallest initial contact angle, whereas oranges display the largest. This indicates that bananas have better wetting properties compared to oranges. This difference may be attributed to the presence of a waxy, hydrophobic layer on the surface of oranges [57, 58]. Furthermore, we conducted a comparative analysis of the surface tension among the aqueous solutions of ACMs, PE, and PE‐CMCS, as illustrated in Figure 5b. The incorporation of CMCS into the coating solution led to a substantial reduction in surface tension, thereby enhancing the adhesion and spreadability of the coating on the fruit's surface and ensuring uniform coverage. To investigate the washability of the film, we incorporated a fluorescent dye into PE‐CMCS and fabricated film samples with a thickness of 150 µm. As the film gradually dissolved in water, the fluorescent dye dispersed into the aqueous phase, leading to a progressive increase in the absorbance of the solution (Figure 5c). We selected Shine Muscat grapes and emperor bananas as experimental subjects to evaluate the washability and non‐residual properties of PE‐CMCS. The surface of the fruit was coated with PE‐CMCS containing Rhodamine 6G, and upon washing with water, the fluorescence disappeared (Figure 5d; Figure S18), thereby confirming the washability of the coating.

Several representative fruits were selected as models for evaluation to assess the feasibility of coatings for preserving fruit freshness, including three climacteric fruits (bananas and cherry tomatoes) as well as a non‐climacteric fruit (strawberries and grapes) [59]. After an observation period spanning 7 to 12 days, it was noted that the uncoated fruits began to show signs of decay and browning. In contrast, the fruits treated with PE‐CMCS coatings retained a favorable appearance for a period exceeding one week (Figure 5e).

### 2.6 Appearance, Mechanical Properties, and Gas Permeability of PE‐CMCS Coating Film

**FIGURE 6:**
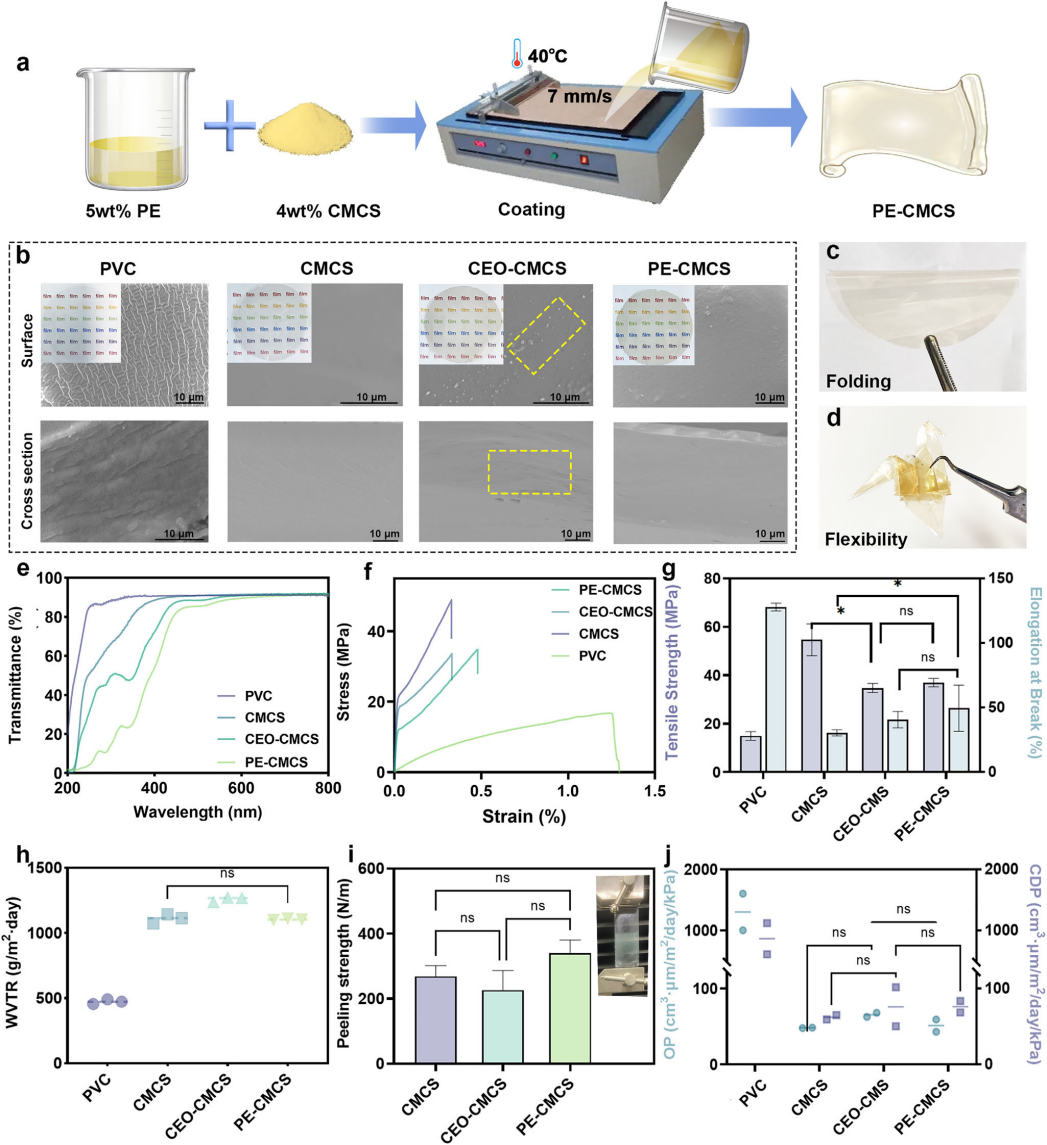
(a) Schematic preparation of PE‐CMCS films. (b) Digital photos and scanning electron microscope images of PVC, CMCS, CEO‐CMCS, and PE‐CMCS films. (c) Photos of PE‐CMCS film showing its foldability. (d) An origami paper crane shows the flexibility of the PE‐CMCS film. Curves of (e) light transmittance, (f) stress‐strain, (g) tensile strength, and elongation at break of PVC, CMCS, CEO‐CMCS, and PE‐CMCS films. (h) The water vapor transmission rate, (i) peeling strength, (j) oxygen permeability (OP) rate, and carbon dioxide permeability rate (CDP) of PVC, CMCS, CEO‐CMCS, and PE‐CMCS films. Data are shown as mean ± SD (*n* = 3), and *p* values were obtained by one‐way ANOVA followed by Duncan's post‐hoc test. Statistical significance is set as *p < 0.05, **p < 0.01, ***p < 0.001, ****p < 0.0001; ns, not statistically significant.

The light transmittance, mechanical properties, and gas permeability of the coating are crucial for its effectiveness in fruit preservation. Therefore, films were prepared through coating, and their physical properties were studied (Figure 6a). As observed, the films containing CEO (PE‐CMCS and CEO‐CMCS) exhibited a pale‐yellow color, as indicated by the positive b* value (Figure 6b; and Table S1, Supporting Information). Moreover, the PE‐CMCS film is flexible and foldable. It can be folded into fragile objects like paper cranes without breaking easily (Figure 6c,d). In contrast, PVC and CMCS films were colorless and transparent, showing higher L* values. Consequently, PVC achieved a transmittance of over 85% across the wavelength range of 250–800 nm (Figure 6e). In comparison, CEO‐CMCS and PE‐CMCS showed reduced transmittance, particularly in the ultraviolet range (200–400 nm). The incorporation of PE endowed the composite film (PE‐CMCS) with excellent ultraviolet‐blocking properties, which can mitigate photo‐oxidation in food products and thereby extend their shelf life. Under the scanning electron microscope, CMCS films displayed a dense and uniform surface morphology (Figure 6b). When CEO was introduced as a blending component into the film‐forming matrix, the surface morphology of CEO‐CMCS became slightly rougher, with small oil droplets observed on the surface (rectangular dashed box). In contrast, the surface of PE‐CMCS is smoother than that of CEO‐CMCS, which can be attributed to the emulsifying effect of ACMs.

Good mechanical properties are essential for a film to function effectively as a fruit coating, enabling it to withstand mechanical stress and prevent premature failure or cracking during processing and storage. We therefore conducted a comprehensive mechanical assessment of the PE‐CMCS film in comparison with commercial PVC film [60, 61], the CMCS matrix, and the CEO‐CMCS film. As shown in Figure 6f,g, the CMCS film exhibited the highest tensile strength (54.65 ± 6.53 MPa). However, it fractured at very low strain (<0.5%), confirming its inherently brittle nature. In contrast, PVC showed a lower tensile strength (14.98 ± 1.79 MPa) but the greatest elongation, consistent with its well‐known ductility. Both CEO‐CMCS and PE‐CMCS films displayed intermediate tensile strengths (30–35 MPa) and significantly improved elongation compared with CMCS (*p* < 0.05). Their stress‐strain curves showed a clear transition from brittle to semi‐ductile behavior, reflecting enhanced structural flexibility while maintaining adequate strength. Furthermore, there was no significant difference in the mechanical properties between PE‐CMCS and CEO‐CMCS, indicating that the addition of Pickering emulsions did not compromise the mechanical integrity of the films.

As shown in Figure 6h, the water vapor transmission rate (WVTR) of PVC (472.44 ± 17.77 g m^−2^ day^−1^) was the lowest, which was consistent with its dense hydrophobic structure (contact angle near 90°) (Figure S19). The WVTR of CMCS was 1110.39 ± 34.39 g m^−2^ day^−1^, consistent with its hydrophilic nature. This value was not significantly different from that of the PE‐CMCS film (1104.99 ± 5.37 g m^−2^ day^−1^; *p* > 0.05). These results indicate that the incorporation of Pickering emulsion did not compromise the water vapor barrier properties. Oxygen and carbon dioxide permeability followed a similar trend (Figure 6j). PVC again had the lowest values, while both CEO‐CMCS and PE‐CMCS films exhibited OP and CDP that were not significantly different from those of CMCS. These findings confirm that the Pickering emulsion retained the intrinsic gas barrier properties of the CMCS matrix without introducing additional porosity or structural defects. The adhesion strength results (Figure 6i) further supported the mechanical stability of PE‐CMCS. The 180° peeling strength of PE‐CMCS showed no significant difference from that of CMCS or CEO‐CMCS, demonstrating that the incorporation of CEO as Pickering droplets did not compromise the coating's interfacial bonding. These results demonstrate that the Pickering emulsion structure did not negatively impact the barrier or adhesion performance of the films, while simultaneously providing additional functional advantages for food preservation.

### 2.7 Preservation Effect and Mechanism of Coatings on Emperor Bananas

**FIGURE 7:**
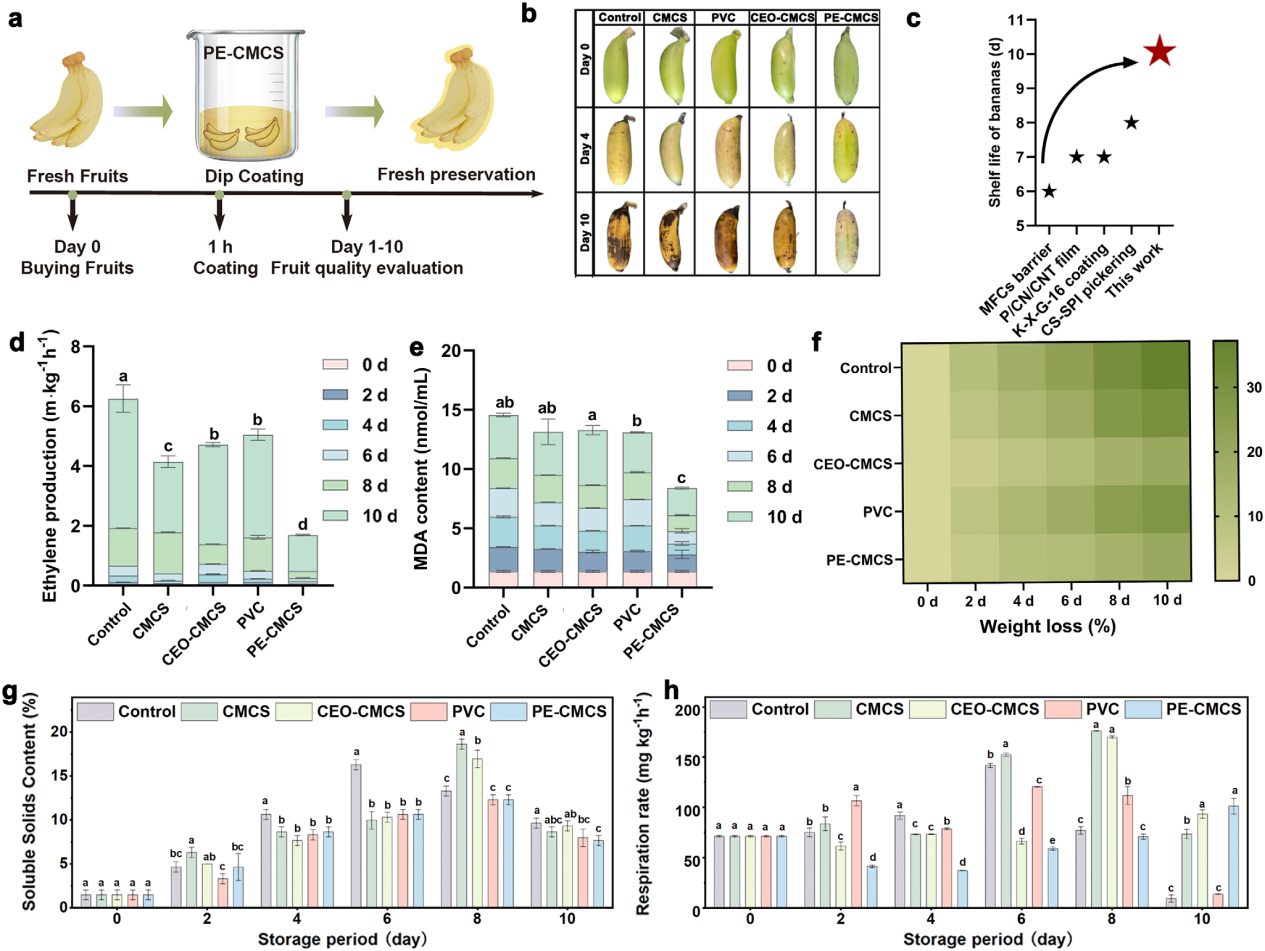
(a) Schematic diagram of coating PE‐CMCS on emperor bananas for fruit preservation. (b) Appearance changes in emperor bananas treated with CMCS, PVC, CEO‐CMCS (CEO directly mixed with CMCS), and PE‐CMCS coating during storage. (c) Comparison of shelf life extension between the PE‐CMCS coating and other reported packaging materials. (d) Ethylene production, (e) MDA content, (f) Weight loss, (g) soluble solids content, and (h) respiration rate of emperor bananas under different treatments. All quantitative data in (d–h) are presented as mean ± SD (*n* = 3). Statistical significance was assessed using one‐way ANOVA followed by Duncan's multiple comparison test (*p* < 0.05). Different letters indicate statistically significant differences among groups.

Inspired by the exceptional antibacterial properties and ethylene adsorption capacity of PE‐CMCS (Figure S20), we desired to explore further its potential for maintaining the physicochemical indicators of fruits. Here, easily perished emperor bananas were chosen as the models, which were dipped into PE‐CMCS solution for 60 s, followed by storage under controlled conditions (25°C ± 0.5°C, 75% relative humidity) for a 10‐day experimental period (Figure 7a). During storage, we systematically assessed various physicochemical parameters, such as weight loss, malondialdehyde (MDA) content, soluble solids content, and ethylene production. Appearance change is the most intuitive way for quality evaluation [62]. Figure 7b shows that uncoated emperor bananas turned dark brown and started to rot by day 10. In contrast, emperor bananas treated with PE‐CMCS retained their fresh appearance. Compared to the shelf life of banana packaging materials developed by other researchers [31, 62, 63, 64], PE‐CMCS coating demonstrated a significantly greater ability, extending the shelf life by up to 10 days (Figure 7c). Polyphenol oxidase (PPO) is also associated with the appearance of the peel, as it produces various melanins that trigger enzymatic browning in fruits [65]. The results revealed that the observed PPO activity levels in the PE‐CMCS group were consistently lower than those in the control group. At the end of storage, the PPO content in the CMCS group was found to be as high as 167.56 ± 1.02 U g^−1^ min^−1^, whereas the PE‐CMCS group exhibited the lowest PPO content at 55.24 ± 0.22 U g^−1^ min^−1^ (Figure S21a). The capturing of endogenous ethylene released by post‐harvest fruits can delay the ripening process of fruits and vegetables [66]. As depicted in Figure 7d, the control group exhibited an ethylene emission rate that peaked at 4.33 ± 0.46 mg kg^−1^ h^−1^ by the tenth day, whereas the rate in the PE‐CMCS group was markedly reduced to 1.20 ± 0.03 mg kg^−1^ h^−1^. The PE‐CMCS film effectively captured ethylene emission, a finding similar to that reported by Li's study [66]. Furthermore, the firmness data (Figure S21b) highlighted the superiority of the PE‐CMCS films. On the tenth day, emperor bananas coated with this film maintained a higher level of firmness compared to those preserved with PVC film.

As shown in Figure 7e, during prolonged storage, a statistically significant difference in MDA content was observed between the control group and the PE‐CMCS group (*p*<0.05). Specifically, the total accumulation of MDA was lower in the PE‐CMCS group, suggesting a reduced extent of cell membrane damage on the fruit surface [7]. Similarly, while unpackaged emperor bananas exhibited a weight loss of up to 37.21 ± 4.83% within 10 days and those wrapped in PVC films lost 28.51 ± 1.05% of their weight over the same period, emperor bananas coated with PE‐CMCS films demonstrated a markedly reduced weight loss of 19.74 ± 2.79% over 10 days (Figure 7f). The pH values of all samples, as depicted in Figure S21c (Supporting Information), exhibit an upward trajectory with the prolongation of storage duration. This ascending trend can be attributed to metabolic activities occurring during storage and the gradual conversion of starch and acids into sugars [67]. Following water evaporation, a progressive reduction in water content within the fruit tissue was observed (Figure S21d), which was consistent with the change in weight loss of emperor bananas.

The soluble solids content (SSC) is regarded as one of the vital parameters to evaluate the fruit postharvest quality [68]. The control group samples reached their highest SSC (16.33 ± 0.58%) by the sixth day, and the PE‐CMCS group peaked (12.33 ± 0.58%) on the eighth day (Figure 7g). The differences were primarily because PE‐CMCS treatment delayed the climacteric respiratory peak of the emperor bananas, with their respiration intensity significantly lower than that of other treatment groups (Figure 7h). The reduction in respiration diminished the consumption of SSC, thereby delaying the senescence and metabolic processes of the fruit [69, 70, 71]. Flame atomic absorption spectrophotometry was used to test whether silver residues were present in the peel and flesh of emperor bananas during the storage period [72]. The average migration amount of Ag^+^ in the bananas was calculated using the standard curve obtained from Figure S22. As shown in Table S2, Ag^+^ was not detected in either the peel or the pulp of the bananas throughout the preservation period. This indicated that coating fruits with PE‐CMCS did not leave residues.

For the PE‐CMCS synthesis, the process required only small amounts of electricity and deionized water and relatively low quantities of ACMs, CMCS, and CEO to produce 0.42 L of coating, which was sufficient to treat 10 kg of emperor bananas, with a total material cost of approximately $6.31 (Table S3). The cost structure was dominated by the ACMs component, whereas the contributions from water, electricity, and CEO were negligible. These indicate clear targets for future cost reduction through process optimization and raw material sourcing. At the ACMs level, life cycle carbon and cost analysis identified electricity consumption and the use of organic solvents such as acetonitrile and isopropanol as the dominant contributors to cost and potential carbon burden. In comparison, the required amount of silver nitrate was extremely low, at only 0.0425 µg/g ACM, and contributed negligibly to the overall mass and cost. This suggests that future improvements should focus on reducing energy intensity and implementing solvent recovery or substitution strategies, rather than on the metal component itself. In parallel, the use of water‐based processing and biodegradable CMCS provides an inherent environmental advantage over conventional petroleum‐based plastic packaging. Given its exceptional safety profile, preservation efficacy, and broad applicability, the PE‐CMCS strategy represents a promising and significant advancement in fruit preservation methods.

### 2.8 Metabolomic Profile of Emperor Bananas Following Coating Treatment

**FIGURE 8:**
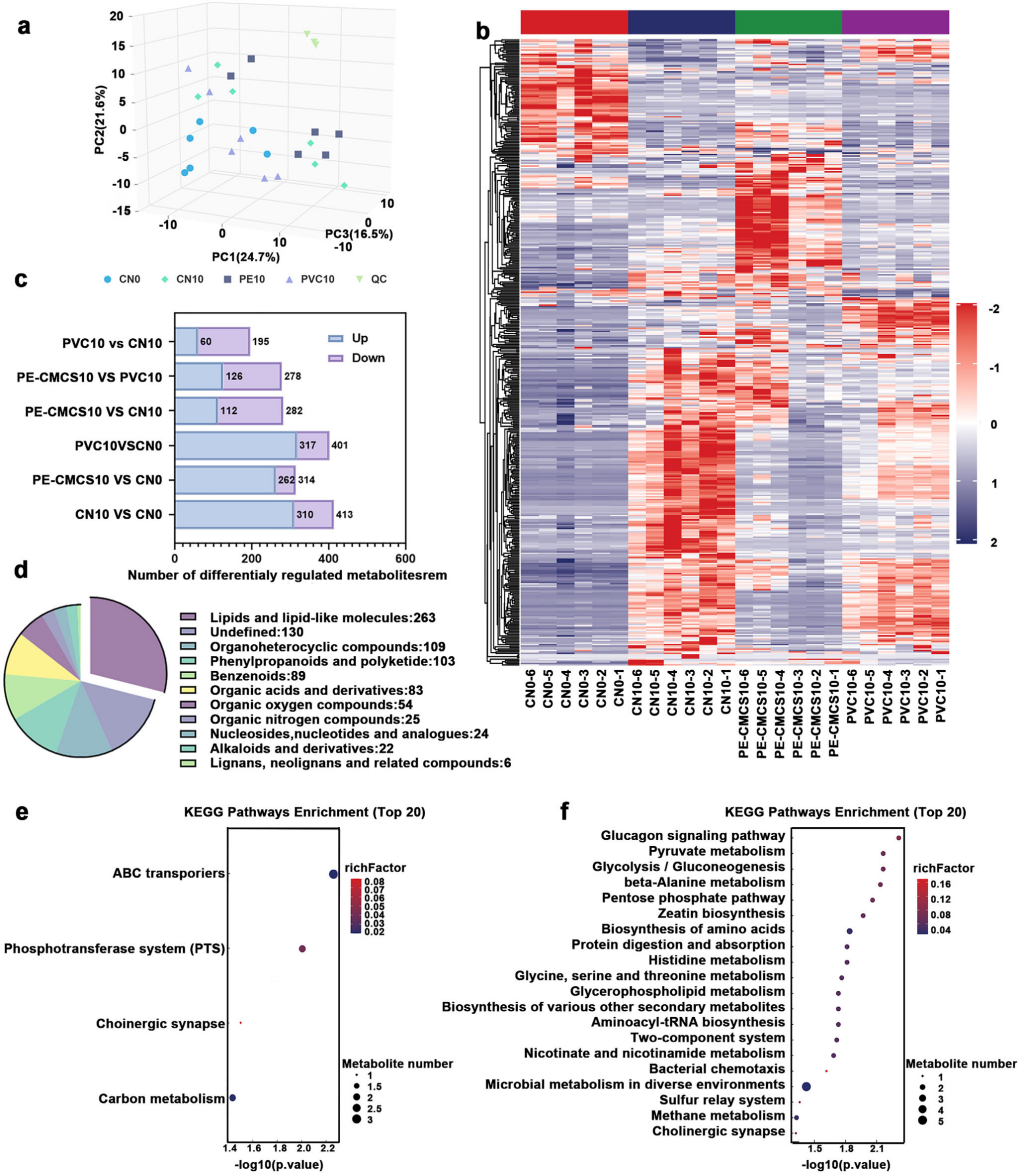
(a) PCA scoring models for all samples in the CN0 (fresh emperor bananas at 0 d), CN10 (emperor bananas stored for 10 d without any treatment), PVC10 (emperor bananas wrapped in PVC cling film and stored for 10 d), and PE‐CMCS10 (emperor bananas treated with PE‐CMCS and stored for 10 d) groups. (b) Clustering heatmap visualization analysis of differential metabolites in CN0, CN10, PVC10, and PE‐CMCS10 groups. (c) Number of up‐ and down‐regulated differential metabolites with CN10 vs CN0, PE‐CMCS10 vs CN0, PVC10 vs CN0, PE‐CMCS10 vs CN10, PE10 vs PVC10, and PVC10 vs CN10. (d) Distribution plot of total metabolite categories. KEGG pathway enrichment analysis of differential metabolites in the groups with (e) PE‐CMCS10 vs CN0 and (f) PVC10 vs CN0.

Metabolomic profiling was conducted to elucidate the mechanism by which the PE‐CMCS coating prolonged fruit preservation. Principal component analysis (PCA) and hierarchical clustering (Figure 8a,b; Figures S23 and S24) revealed distinct metabolic separations among treatment groups (denoted as CN10, PVC10, and PE‐CMCS10 for samples at day 10), indicating that each coating treatment distinctly influenced the fruit's metabolic state. Notably, bananas treated with PE‐CMCS exhibited the fewest metabolic perturbations, with only 314 differentially expressed metabolites identified (Figure 8c; Figure S25), compared to 413 and 401 in the CN10 and PVC10 groups, respectively. This suggested that the PE‐CMCS coating significantly mitigated the metabolic transitions associated with fruit senescence. These differentially expressed metabolites were primarily enriched in classes such as organic oxygen compounds, lipids, and benzenoids (Figure 8d), which play critical roles in ripening. By modulating the abundance of these compounds, the PE‐CMCS coating appeared to slow the senescence process.

KEGG enrichment analysis further corroborated these findings. The PE‐CMCS10 group exhibited significant enrichment in only four metabolic pathways, markedly fewer than those observed in CN10 (16 pathways) and PVC10 (17 pathways) (Figure 8e,f; Figure S26). The scarcity of significantly altered pathways indicated that PE‐CMCS exerted the least disruption on the global metabolic network. These pathways primarily included carbon metabolism, transport systems, and signaling‐related processes. This indicated that PE‐CMCS exerted the least interference with key metabolic networks. Notably, metabolic pathways related to glycolysis, starch and sucrose metabolism, and amino acid catabolism showed fewer changes in the PE‐CMCS group. These pathways are commonly affected by ethylene accumulation during fruit ripening. The alterations observed here were consistent with those previously reported [73]. This evidence further supports the role of the PE‐CMCS coating in modulating ethylene‐associated metabolic processes.

Phospholipids such as phosphatidylcholine typically increase during membrane degradation. In this study, their levels rose markedly in both the CN10 and PVC10 groups. However, no such elevation was observed in the PE‐CMCS‐treated samples (Figures S27–S29) [74]. These findings suggest that PE‐CMCS helped maintain membrane stability during storage. Furthermore, amino acids linked to aroma formation, including valine, accumulated more slowly in the PE‐CMCS10 group compared to CN10 and PVC10 [75], indicating delayed ripening. Collectively, these results demonstrated that the PE‐CMCS coating effectively modulated metabolic pathways associated with senescence, thereby extending the shelf life of emperor bananas.

## 3 Conclusions

In summary, a MOF‐stabilized Pickering emulsion (PE) was proposed as a smart coating for efficient preservation of perishable fruits. Through a simple dipping process, PE‐CMCS formed a uniform and washable protective film on the fruit peel surface. This film effectively absorbed endogenous ethylene released by the fruit, inhibited microbial contamination, and significantly extended the shelf life of the fruit. Moreover, PE‐CMCS delayed the significant accumulation of differential metabolites, such as valine, phosphatidylethanolamine, and phosphatidylcholine, thereby reducing nutrient depletion. This approach offered innovative solutions for antimicrobial coatings and ethylene adsorption applications.

## 4 Experimental Section

### 4.1 Statistical Analysis

All statistical analyses were conducted using SPSS 26.0 (IBM, USA), GraphPad Prism 9.0 (GraphPad Software, USA), and Origin 2021 (OriginLab, USA). Data were examined for quality before analysis. Data preprocessing included outlier evaluation and normalization, with results expressed as mean ± SD. Sample sizes were determined by experimental design and are reported in the corresponding figure captions or methods. For comparisons among multiple groups, one‐way ANOVA was performed using two‐sided testing at a significance level of α = 0.05, followed by Duncan test for pairwise analysis. Metabolomics data were analyzed using multivariate statistics, including PCA, PLS‐DA, volcano plot analysis, and KEGG pathway enrichment, following established metabolomics workflows. Statistical significance was defined as *p* < 0.05.

Full reagents and experimental procedures were provided in the Supporting Information. [CCDC 773709 contains the supplementary crystallographic data for this paper. These data can be obtained free of charge from The Cambridge Crystallographic Data Centre via www.ccdc.cam.ac.uk/data_request/cif.].

## Ethics Statement

Kunming Mice (male, 4–6 weeks old) were purchased from SPF Biotechnology Co. Ltd., and all experiments were approved by the Ethics Committee of Inner Mongolia University (the assigned approval/accreditation number: IMU‐2023/037).

## Conflicts of Interest

The authors declare no conflicts of interest.

## Supporting information




**Supporting File**: advs73585‐sup‐0001‐SuppMat.docx.

## Data Availability

The data that support the findings of this study are available from the corresponding author upon reasonable request.
