# Supplementary material for: Efficient Preservation of Perishable Fruits by Erasable Metal‐Organic Frameworks
Source: Adv Sci (Weinh). 2026 Jan 4;13(14):e19222. doi: 10.1002/advs.202519222 (PMC12970200; doi:10.1002/advs.202519222)
Supplement: Supplementary file 1 — Supporting File: advs73585‐sup‐0001‐SuppMat.docx. [file ADVS-13-e19222-s001.docx]

Supporting Information

**Efficient Preservation of Perishable Fruits by Erasable Metal-Organic Frameworks**

*Liying Yang, Jia Kong, Haoran Bai, Xiaojie Wu, Douxin Xiao*, Ming Du*, Peng Yang*, Alideertu Dong**

L. Yang, H. Bai, X. Wu, D. Xiao, A. Dong

Engineering Research Center of Dairy Products Quality and Safety Control Technology, Ministry of Education, College of Chemistry and Chemical Engineering, Inner Mongolia University, Hohhot 010021, P. R. China

E-mail: xiaodouxin@imu.edu.cn (D. Xiao), dongali@imu.edu.cn (A. Dong)

J. Kong, P. Yang

Key Laboratory of Applied Surface and Colloid Chemistry, Ministry of Education, School of Chemistry and Chemical Engineering, Shaanxi Normal University, Xi'an 710119, P. R. China

1. mail: yangpeng@snnu.edu.cn (P. Yang)

M. Du

School of Dalian Ocean University, Dalian 116000, P. R. China

E-mail: duming121@163.com (M. Du)

**Table of contents**

[Table of contents 2](#_Toc32105)

[1. Materials and Methods 3](#_Toc20320)

[In situ synthesis of ACMs 3](#_Toc6794)

[Preparation of PE solution 4](#_Toc7756)

[Preparation of PE-CMCS coating solution 4](#_Toc10737)

[Fourier transform infrared (FTIR) spectroscopy 4](#_Toc11828)

[X-ray powder diffraction (XRD) 5](#_Toc10246)

[X-ray photoelectron spectroscopy (XPS) 5](#_Toc29228)

[Scanning electron microscope (SEM) 5](#_Toc19096)

[Transmission electron microscopy (TEM) 5](#_Toc17325)

[Cry-electron microscopy 5](#_Toc7313)

[Dynamic light scattering (DLS) 5](#_Toc9191)

[Optical microscope (OM) 6](#_Toc31889)

[Rheological properties 6](#_Toc30848)

[Thermal stability and specific surface area 6](#_Toc15646)

[BET surface area and pore size 6](#_Toc10102)

[Antimicrobial properties 6](#_Toc23427)

[Grand Canonical Monte Carlo 7](#_Toc21257)

[Biocompatibility testing of PE-CMCS coating 8](#_Toc10915)

[In vivo biosafety analysis 9](#_Toc15814)

[Physical property test of PE-CMCS coating 9](#_Toc31035)

[Physical property test of PE-CMCS films 11](#_Toc24628)

[Preservation efficacy of coating on bananas 11](#_Toc14191)

[Soluble solids content (SSC) 11](#_Toc17592)

[Water content 11](#_Toc32158)

[Polyphenol oxidase content 12](#_Toc9117)

[Malondialdehyde content 12](#_Toc9918)

[Respiratory rate 12](#_Toc18540)

[Migration test 13](#_Toc737)

[Metabolomics determination and analysis of emperor bananas 14](#_Toc21238)

[Statistical Analysis 15](#_Toc2699)

[2. Supplementary Figures 16](#_Toc14783)

[3. Supplementary Tables 45](#_Toc508)

[4. Supplementary References 50](#_Toc24746)

**1. Materials and Methods**

The γ-CD (98%) and AgNO_3_ were supplied by Shanghai Macklin Biochemical Co., Ltd. Octadecenylsuccinic anhydride (ODSA) was purchased from Shanghai Macklin Biochemical Co., Ltd. Clove essential oil (CEO) was bought from Shanghai Jingchun Biotechnology Co., Ltd. Carboxymethyl chitosan was provided by Xian Qiuhe Biotechnology Co., Ltd. Isopropyl alcohol and acetonitrile were obtained from Tianjin Fuchen Chemical Reagent Technology Co., Ltd.

**Preparation of OCMs**

The γ-CD was uniformly dispersed in water to form a stable suspension. Subsequently, the pH of the suspension was adjusted to 8.5 using an aqueous solution of 1% NaOH. Gradual addition of ODSA to the aqueous γ-CD solution ensured complete dissolution. Once the pH value reached a constant level, indicating reaction completion, it was adjusted to 6.5 using a 1 mol/L HCl solution. The resulting solution then underwent freeze-drying treatment followed by washing with a hexane/isopropanol mixed solvent (3:1 v/v). Finally, the washed product was vacuum dried at 40℃ overnight to obtain modified γ-CD powder.

A solution was prepared by dissolving 1.296 g of hydrophobically modified γ-CD and 0.448 g of potassium hydroxide in 40 mL of distilled water. The resulting mixture was filtered through a 0.8 μm hydrophilic filter membrane and transferred to a small beaker. Subsequently, 4 mL of methanol was added to the solution. The small beaker was then placed inside a larger beaker containing 32 mL of methanol. Crystals were grown via the diffusion of methanol vapor into the solution under a constant temperature bath at 50°C for 6 h. After the reaction was completed, the liquid in the small beaker was collected, and an equal volume of methanol and 0.18 mg of CTAB were added. The mixture was then incubated at room temperature for 24 h. The precipitate was subsequently collected by centrifugation at 8000 rpm for 10 min, followed by washing with isopropanol three times. Finally, the product was vacuum-dried overnight at 50°C.^[1]^

**In situ synthesis of ACMs**

For the synthesis of ACMs, a combined method involving solvent impregnation and reactive diffusion was utilized.^[2]^ Initially, OCMs (600 mg) were immersed in acetonitrile (1.5 mL) and allowed to equilibrate for 72 h. Subsequently, the crystals were subjected to a soaking process in an acetonitrile solution of AgNO_3_ (10 mM) for 72 h. This extended soaking period enabled the gradual reaction and diffusion of Ag ions into the interior framework of the OCMs. The resultant precipitate was isolated via high-speed centrifugation at 10000 rpm. Finally, the precipitate was vacuum-dried overnight at 40°C.

**Preparation of PE solution**

The CEO was initially mixed with the ACMs, which had been previously dissolved in ultrapure water. Subsequently, the mixture was thoroughly emulsified using a homogenizer operating at 12000 rpm for 3 min. Cyclodextrin solutions of various concentrations (1.25 wt%, 2.5 wt%, 5 wt%, and 10 wt%) were prepared to evaluate their influence on PE. Additionally, different oil/water volume ratios (1:99, 1:19, 1:9, and 3:7) were employed to further investigate their effects on PE.

**Preparation of PE-CMCS coating solution**

Carboxymethyl chitosan (CMCS) was dissolved in water to obtain a 40 mg/mL solution. The mixture was heated at 50°C for 2 h and continuously stirred at 500 rpm on a magnetic stirrer. The resulting CMCS solution was then mixed with PE at a volume ratio of 1:19 and mechanically stirred at 1000 rpm for 30 min at room temperature, producing the PE-CMCS coating solution.

PE-CMCS films were prepared by dispensing the coating solution onto the platform of a coating machine and applying it with a wire-bar coater at 40 °C and a coating speed of 7 mm/s. The coated layer was subsequently left to stand and dry to form films for further experiments.

**Fourier transform infrared (FTIR) spectroscopy**

A thin section was prepared using the pressing technique by compressing a mixture of potassium bromide and the powdered sample. Potassium bromide served as the background material for Fourier Transform Infrared (FTIR) spectroscopy measurements. The sample was scanned within the wavenumber range of 400 to 4000 cm⁻¹ using an FTIR spectrometer (NICOLET6700, Thermo Fisher, Massachusetts, USA) to record its infrared spectral signature.

**X-ray powder diffraction (XRD)**

In order to gain insights into the phase structure of the crystals obtained, a 20 mg aliquot of the crystal was carefully weighed and subjected to XRD (Empyrean, PANalytical B.V., Almelo, Holland) analysis. This was conducted with a scanning speed of 2°/min, targeting a diffraction angle 2θ that ranges from 5° to 30°.

**X-ray photoelectron spectroscopy (XPS)**

XPS (ESCALABXi+, Thermo Fisher, Massachusetts, USA) was employed to assess the elemental states present at the surface of the sample. The acquired data were then analyzed using the Advantage software.

**Scanning electron microscope (SEM)**

For the SEM observations, the samples were coated with a 50 nm thick gold layer. The morphology of CMs, OCMs, and ACMs was observed under an SEM (SSX-550, Shimadzu Corp, Japan).

**Transmission electron microscopy (TEM)**

TEM experiments were conducted on an HT 7800 electron microscope (Hitachi, Tokyo, Japan). The ACMs were characterized using transmission electron microscopy (TEM) fitted with a tungsten filament, giving a point resolution of 0.2 nm.

**Cry-electron microscopy**

The microscopic morphology of the PE was characterized using a Hitachi SU8100 cryogenic scanning electron microscope. The PE sample was rapidly plunge-frozen for 30 seconds in liquid nitrogen slush to preserve its native structure. Subsequently, a low-temperature cryogenic preparation and transfer system was utilized to transfer the sample under vacuum conditions into the sample preparation chamber for sublimation gold coating.

**Dynamic light scattering (DLS)**

To prepare the samples for testing, ultrapure water was used as the dispersing medium to dilute the samples to a concentration of 0.02 g/mL, followed by sonication to ensure homogeneous distribution of the particles. The particle size distribution and Zeta potential of the samples were then measured using the Zetasizer Pro instrument (Malvern Panalytical, Malvern, UK).

**Optical microscope (OM)**

The microstructure of the PE was observed using OM (DM2500, Leica, Weizler, Germany), with a focus on examining the emulsification degree of the emulsion.^[3]^

**Rheological properties**

The rheological properties of the PE were characterized using a rheometer (MCR, Anton Paar, Graz, Austria). A parallel aluminum plate fixture with a diameter of 20 mm was employed, and the shear rate range was set from 0.1 to 100 s^-1^. Changes in the apparent viscosity of the emulsion were recorded throughout the experiment. During the dynamic frequency sweep, the frequency was varied between 0.1 and 10 rad/s with a constant strain of 1%. The variations in storage modulus (G') and loss modulus (G") with frequency were recorded to gain a deeper understanding of the viscoelastic properties of the material. All tests were carried out at a constant temperature of 25°C.^[4]^

**Thermal stability and specific surface area**

TGA (STA449 FJupiter, Netzsch, Selbu, Germany) was used to test the thermogravimetric weight of different samples. Under the protection of nitrogen, the temperature increase rate was 10 °C/min, and the heating interval was from room temperature to 800°C.

**BET surface area and pore size**

Nitrogen adsorption-desorption isotherms were measured using a specific surface area and porosity analyzer (ASAP 2260, Micromeritics, Atlanta, USA). Before analysis, the samples were treated by soaking in dichloromethane for three days, followed by vacuum drying to yield solid samples. Subsequently, the samples were degassed at 100°C for 6 h and analyzed under an N_2_ atmosphere at 77 K.^[5]^

**Antimicrobial properties**

Utilizing a contact sterilization assay to evaluate the antibacterial efficacy of samples.^[6]^ Specifically, nutrient agar was evenly poured onto the surface of dishes to establish an optimal growth environment. Subsequently, bacterial suspensions at a concentration of 10^7^ CFU/mL were individually treated with equal concentrations of CEO, ACMs dissolved in water, and PE via contact sterilization. After treatment, the suspensions were uniformly spread onto the surfaces of the petri dishes. Each condition was prepared in triplicate. The antibacterial activity was assessed using the plate count method.

We employed the NIKON AIR confocal laser scanning microscope (CLSM) to evaluate alterations in bacterial membrane permeability.^[7]^ Initially, the bacteria were stained with a dual stain of SYTO9 and PI. Subsequently, the bacterial suspension was harvested and washed three times with phosphate-buffered saline (PBS) to minimize background fluorescence and enhance the accuracy. Lastly, the stained bacteria were examined under the CLSM across three distinct microscopic fields.

In the fungal inhibition experiment, potato dextrose agar (PDA) was used as the basal medium for culturing the fungi. For the experimental groups, PDA was supplemented with CEO, ACMs, and PE. The colonies of *Botrytis cinerea* were inoculated onto these media and incubated in a growth chamber at 28°C for 10 days. *Penicillium italicum* was similarly inoculated onto the culture medium and incubated at 28°C for 15 days, whereas *Penicillium digitatum* was inoculated onto the medium and incubated at 25°C for 7 days. Colony diameters were measured every 5 days using a caliper gauge, and photographs were taken to record growth patterns. Finally, inhibition rates were calculated based on the colony diameter measurements.

When examining the surface morphology of hyphae in fungi and bacteria, it is essential to fix the samples overnight using glutaraldehyde. This is followed by a series of ethanol gradient dehydration treatments with increasing concentrations of 25%, 50%, 70%, 90%, and 95% ethanol. Specifically, for fungal samples, after completing the ethanol gradient dehydration process, additional dehydration is required using isoamyl acetate to replace the dehydrated ethanol. Subsequently, the samples undergo lyophilization before being analyzed by scanning electron microscopy.

**Grand Canonical Monte Carlo**

We employed the Sorption module within the Materials Studio 2022 software to simulate the ethylene adsorption process by ACMs. The core of the Sorption module lies in the application of the Grand Canonical Monte Carlo (GCMC) method, which enables precise simulation of the adsorption process. During the simulation, we selected the COMPASS force field within the Sorption module to comprehensively describe the interaction between ethylene and ACMs. The cut-off radius of the LJ potential is selected as 12.5 Å. The interaction energies *E_in_* between MOF and ethene molecules were calculated according to the following equation (1)

$E_{\mathrm{in}}=E_{\mathrm{total}}-E_{\mathrm{MOF}}-E_{\mathrm{etheme}}$ (1)

where *E_total_* is the total energy of the ethene sorption MOF, *E_MOF_* is the energy of MOF (CMs or ACMs), and *E_ethene_* is the energy of ethene molecules in MOF.

**Biocompatibility testing of PE-CMCS coating**

Hemolysis assays were performed to evaluate the hemocompatibility of the PE-CMCS coating. Whole blood was collected from mouse eyes, stored in anticoagulant tubes at 4°C, and centrifuged at 1500 rpm for 10 min to remove serum. RBCs were washed three times with 0.9% saline to obtain pure samples. The positive control (Triton X-100) and negative control (0.9% saline) were prepared along with experimental groups containing leachate, CEO, or ACMs. After incubation at 37°C for 30 min, the samples were centrifuged at 3500 rpm for 10 min, and the absorbance was measured at 540 nm. The hemolysis rate was calculated using formula (2).

$Hemolysis rate\left（ \% \right）=\frac{\mathrm{OD}_{\mathrm{sample}}-\mathrm{OD}_{\mathrm{negative}}}{\mathrm{OD}_{\mathrm{positive}}-\mathrm{OD}_{\mathrm{negative}}}\times100\%$ (2)

NIH-3T3 cells and Caco-2 cells were seeded in a 96-well plate (100 μL DMEM per well) and cultured at 37°C with 5% CO_2_ for 24 h. After reaching confluence, the medium was replaced with 100 μL of medium containing leachate. After a further incubation of 24 h, 10 μL CCK-8 dye was added to each well. After 1 hour, OD values were measured at 450 nm, and cell viability was calculated using equation (3).

$Cell survival rates \left（ \% \right）=\frac{\mathrm{OD}_{\mathrm{sample}}-\mathrm{OD}_{\mathrm{control}}}{\mathrm{OD}_{\mathrm{control}}-\mathrm{OD}_{\mathrm{blank}}}\times100\%$ (3)

OD_sample_ refers to the OD value of the experimental group, the OD_control_ refers to the OD value of cells without any treatment, and the OD_blank_ refers to the OD value of the medium.

The cell viability of PE-CMCS film leachates was assessed using a cell viability/toxicity assay kit. NIH-3T3 cells were seeded at equal densities in six-well plates, each well containing 1 mL of DMEM medium. Cover slips were placed in the wells, and the cells were cultured in a 37°C incubator with 5% CO_2_ until the cover slips were completely covered with cells. The medium was then discarded, and the cells were treated with prepared PE-CMCS leachate at concentrations of 5 cm^2^/mL, 1 cm^2^/mL, 1.5 cm^2^/mL, and 2 cm^2^/mL. After 24 h of incubation in a 37°C incubator with 5% CO_2_, the leachate was removed. Subsequently, 1 mL of PBS dye containing 0.5% propidium iodide (PI) and 0.5% acetoxymethyl ester (AM) was added to each well, and the cells were incubated for 1 hour in the dark. The coverslips were then removed, placed on microscope slides, and sealed for machine testing.^[8]^

An apoptosis detection kit was used to assess apoptosis in NIH-3T3 cells. The adherent cells were digested with trypsin, and the detached cells were transferred to a centrifuge tube. After centrifugation to remove the supernatant, the cells were resuspended in 1 mL of PBS, and the supernatant was discarded. The cells were then resuspended in 100 μL binding buffer at a concentration of 1–5·10^6^ cells/mL. Then, 5 μL FITC dye was added, and the mixture was incubated for 5 min in the dark at room temperature. Then, 5 μL PI dye and 400 μL PBS were added, and the solution was homogenized before being subjected to automated analysis.

**In vivo biosafety analysis**

Kunming mice (male, 4–6 weeks) were obtained from the Spearfish (Beijing) Biotechnology Co., Ltd., and all animal experiments were approved by the university’s Institutional Review Board (IMU-2023/037). Animal husbandry was performed following the Ministry of Health of the People’s Republic of China’s *Guidelines on Animal Management and the Chinese Guide for the Use of Laboratory Animals in Husbandry*. After 3 days of environmental adaptation, the mice were randomly divided into the treatment group (PE-CMCS, dose 2 mg/kg) and the normal saline group (Control), with 6 mice in each group for 14 days. The body weight of mice in each group was measured and recorded at fixed time points every day. Fourteen days later, the mice were sacrificed, and the main organs, including the heart, spleen, liver, kidney, and lung, were taken for H&E staining and blood routine analysis.

**Physical property test of PE-CMCS coating**

First, 4 g of CMCS were dissolved in 100 mL of water. Subsequently, the prepared PE emulsion was mixed with the CMCS solution at a volume ratio of 1:19 using mechanical stirring, resulting in a coating solution for subsequent physical property tests.

The surface tension was measured at room temperature using the platinum plate method. The tensiometer was powered on 30 min before the experiment to allow sufficient warm-up and to ensure measurement stability. Before each test, the platinum plate was tilted at 45° and flame-treated with an alcohol burner until it reached a dull red color, removing any surface contaminants. Subsequently, 10 mL of the PE-CMCS solution was transferred into the sample dish, and the instrument was adjusted to lower the plate slowly toward the liquid surface. When the plate became parallel to the surface and made initial contact with the liquid, the maximum force generated by capillary action was recorded. This value was taken as the surface tension of the solution.

To test the water solubility of the coating, Rhodamine 6G fluorescent dye was mixed into the PE-CMCS coating solution. After mixing evenly, the coating solution was poured onto a polytetrafluoroethylene plate and left to form a film overnight. The film was then placed in a sample bottle containing deionized water to observe changes in the film. Every 2 min, 1 mL of deionized water was sampled from the bottle to test its UV absorption spectra and to photograph the changes in the film within the sample bottle.

The washability of the coating was assessed by adapting the method reported by Jung et al.^[9]^ A total of 1 mL of a 5% rhodamine 6G solution was added to 100 mL of the PE-CMCS coating solution and mixed at 1000 rpm using a magnetic stirrer at room temperature until homogeneous. The fluorescent coating solution was then applied to the surfaces of Shine Muscat grapes and emperor bananas by dip coating. After drying naturally at room temperature, the coated fruits were rinsed with 100 mL of water for 2 min using a constant-pressure dropping funnel to maintain a uniform flow rate. Slices were subsequently excised from the fruit surfaces and examined using confocal laser scanning microscopy (CLSM). This group was designated as the washed group. The “uncoated” consisted of slices from fruits without any treatment, whereas the “coated” included slices from fruits coated with the fluorescent solution but not subjected to rinsing.

**Physical property test of PE-CMCS films**

The appearance performance of the film was evaluated by a Minolta colorimeter. After liquid nitrogen pretreatment of the film, its surface and cross-sectional microstructure were observed by scanning electron microscope (SEM). The light transmittance of the film was determined by a ultraviolet-visible spectrophotometer. The water vapor transmission rate was measured using a water vapor transmission rate tester (W3-031) from Jinan Labthink Mechatronics Technology Co., and the oxygen and carbon dioxide transmission rates were determined by a gas transmission rate tester of Jinan Sike Testing Technology Co., Ltd. The mechanical properties of the film were evaluated by the UTM2502 universal material testing machine produced by Shenzhen Sansi Zongheng Technology Co., Ltd.

**Preservation efficacy of coating on bananas**

The bananas used in this study were selected based on their equal ripeness, similar size and color, and absence of mechanical damage to their surfaces. The commercial quality of bananas was evaluated through various indices, namely, hardness, weight, soluble solids content, water content, pH value, ethylene, polyphenol oxidase (PPO), and malondialdehyde (MDA). Bananas were stored in a room temperature incubator at 25°C to simulate the picking and transportation conditions.

Firstly, the banana samples were weighed daily to calculate the rate of weight loss.^[6]^ In addition, the hardness of the bananas was measured using a durometer (GY-1, Sanliang, Dongguan, China), selecting the middle part of each banana and taking the average.^[10]^

**Soluble solids content (SSC)**

For the soluble solids content (SSC), the banana pulp was ground and filtered, and then measured with a refractometer (SN-DR-3201, Shangpu, Shanghai, China). This procedure was repeated in triplicate to obtain an average value.^[11]^

**Water content**

The water content of bananas can be calculated through the weight variation before and after freeze-drying, utilizing the formula Eq. (4).

$Water content (\%)=\frac{m_{1}-m_{2}}{m_{1}}\times100$ (4)

where m_1_ is the weight of the banana before freeze-drying (g), and m_2_ is the weight of the banana after freeze-drying (g).

**pH measurement**

For banana samples treated with different methods, the juice was extracted by grinding, squeezing, and filtering. The pH changes were measured using a pH meter, and the process was repeated three times to obtain an average.

**Polyphenol oxidase content**

The bananas were ground to a pulp, and the polyphenol oxidase (PPO) assay kit (A136, Jiancheng, Nanjing, China) was used to determine the changes in PPO content. The instructions for use were strictly adhered to throughout the process. The absorbance at 460 nm was measured to calculate the changes in PPO content.

**Malondialdehyde content**

The banana was ground into pulp and used in conjunction with the malondialdehyde (MDA) test kit (A003, Jiancheng, Nanjing, China). The instructions for use were strictly followed throughout the procedure. The absorbance at 532 nm was measured, and the change in MDA content was calculated.

**Respiratory rate**

The respiratory rate was measured using the method according to Liu et al.^[12]^ Specifically, each treated banana was placed in a 2L sealed container and incubated at a constant temperature of 20°C for 20 min. Subsequently, the use of a pump-suction gas detector (HCK2000, Krypton, Shenzhen, China) allowed for the insertion of the needle into the sealed container, enabling the measurement of carbon dioxide and ethylene production. The whole gas sampling process took 15 seconds, and the results were expressed in ppm. Breathing rate and ethylene production were calculated using Eq. (5).

$R=\frac{(w_{2}-w_{1})\times V\times M}{V_{0}\times m\times t}$ (5)

where R was the respiration intensity, mg kg^−1^·h^−1^; w_1_ was CO_2_ original concentration, ppm; w_2_ was CO_2_ concentration at the end of the reaction, ppm; V was the total volume of the breathing chamber, L; M was the molar mass of CO_2_, g·mol^−1^; V_0_ was the molar volume of CO_2_, L·mol^−1^; m was the mass of the sample, g; t was determination time, h.

**Migration test**

In order to prepare a series of silver standard solutions, start by diluting 1 mL of a silver standard solution (1000 mg/L) to 100 mL to obtain an intermediate solution with a concentration of 10 mg/L. Then prepare a series of silver standard solutions by taking 0 mL, 1 mL, 2 mL, 4 mL, 8 mL, and 10 mL of the intermediate solution and diluting each to 100 mL. This will give a series of solutions with concentrations between 0 and 1 mg/L. The absorbance of these solutions should then be measured in order of increasing concentration using a flame atomic absorption spectrophotometer.

Immerse the emperor banana in a PE-CMCS coating solution for 10 seconds, and after drying, grind the peel and pulp separately. Take 0.5 g of the ground pulp and place it into a digestion vessel, add 5 mL of nitric acid for pre-digestion at 130°C for 80 min. After cooling, add 3 mL of nitric acid and let it stand for 10 min. Ramp up to 120°C in 5 min, and hold at that temperature for 5 min; then ramp up to 160°C in another 5 min, hold for 10 min; finally, ramp up to 180°C in the last 5 min, and hold for 10 min. After digestion, cool and drive off the acid until the volume is reduced to 1 mL. Transfer to a 50 mL volumetric flask, wash the digestion vessel, and make up to volume, then measure the absorbance. The blank control is an untreated Emperor banana. Subsequently, load the samples for analysis, in triplicate, take the average of the absorbance values, and calculate the silver content (X) in the emperor banana using Eq. (6).

$X=\frac{(\rho-\rho_{0})\cdot V}{m}$ (6)

where ρ is the mass concentration of silver in the sample solution, with units of mg/kg; ρ_0_ is the content of silver in the pattern of the blank group, measured in mg/kg; V is the final volume of the sample digestion solution, measured in mL. m is the mass of the sample, measured in grams.

**Metabolomics determination and analysis of emperor bananas**

The samples were collected on the 1st and 10th days after storage, respectively. Approximately 1 g of emperor banana pulp was weighed into a 50 mL centrifuge tube and sealed. After subjecting the samples to liquid nitrogen treatment, they were stored in a -80℃ freezer for subsequent use, with six biological replicates for each group. Following slow thawing at 4℃, an appropriate amount of sample was extracted and added to a pre-cooled solution of methanol/acetonitrile/water (2:2:1, v/v). The mixture was vortexed and sonicated at low temperature for 30 min, followed by incubation at −20℃ for 10 min. Subsequently, the samples were centrifuged at 14000 g for 20 min at 4°C. The supernatant was then subjected to vacuum drying, and reconstitution during mass spectrometry analysis involved adding 100 μL of acetonitrile/water solution (acetonitrile: water = 1:1, v/v). The mixture was vortexed again and centrifuged at 14000 g for 15 min at 4°C before collecting the supernatant for injection and analysis.

The separation was performed using a C18 column maintained at a temperature of 40℃ and a flow rate of 0.4 mL/min. The injection volume was set at 2 μL. The mobile phase consisted of two components: A, which comprised water with 25 mM ammonium acetate and 0.5% formic acid, and B, methanol. The gradient elution program proceeded as follows: initially, the proportion of component B was held at 5% from 0 to 0.5 min; subsequently, the concentration of component B increased linearly from 5% to reach 100% between 0.5 and 10 min; This concentration was subsequently maintained at a constant level of 100% throughout the time interval ranging from 10 to 12 min; following this period, the concentration of component B rapidly decreased from its maximum value (100%) to return to an initial level (5%) within the span between 12 and 12.1 min; finally, the concentration of component B remained stable at a fixed percentage (5%) during the time interval spanning from 12.1 up until 16 min. Throughout the analysis, samples were maintained at a temperature of 4℃ in an automated sampler. In order to mitigate the impact of instrument signal fluctuations, samples were consecutively analyzed in a randomized sequence. Quality control (QC) samples were incorporated into the sample queue to monitor and assess both system stability and experimental data reliability.

**Statistical Analysis**

All statistical analyses were conducted using SPSS 26.0 (IBM, USA), GraphPad Prism 9.0 (GraphPad Software, USA), and Origin 2021 (OriginLab, USA). Data were examined for quality before analysis. Data preprocessing included outlier evaluation and normalization, with results expressed as mean ± SD. Sample sizes were determined by experimental design and are reported in the corresponding figure captions or methods. For comparisons among multiple groups, one-way ANOVA was performed using two-sided testing at a significance level of α = 0.05, followed by Duncan test for pairwise analysis. Metabolomics data were analyzed using multivariate statistics, including PCA, PLS-DA, volcano plot analysis, and KEGG pathway enrichment, following established metabolomics workflows. Statistical significance was defined as *p* < 0.05.

1. **Supplementary Figures**


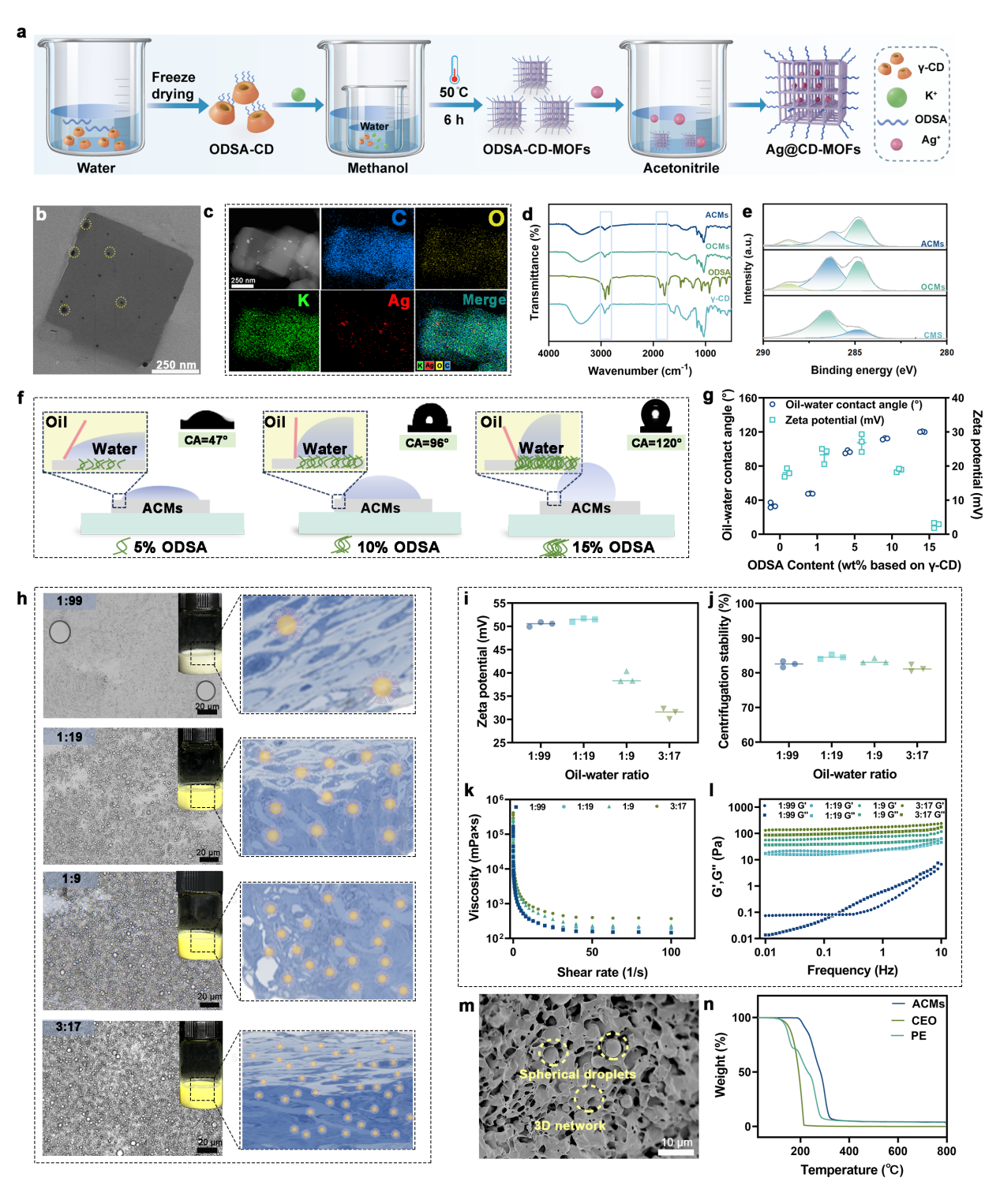


**Figure S1.** Schematic illustration of the preparation of ACMs.


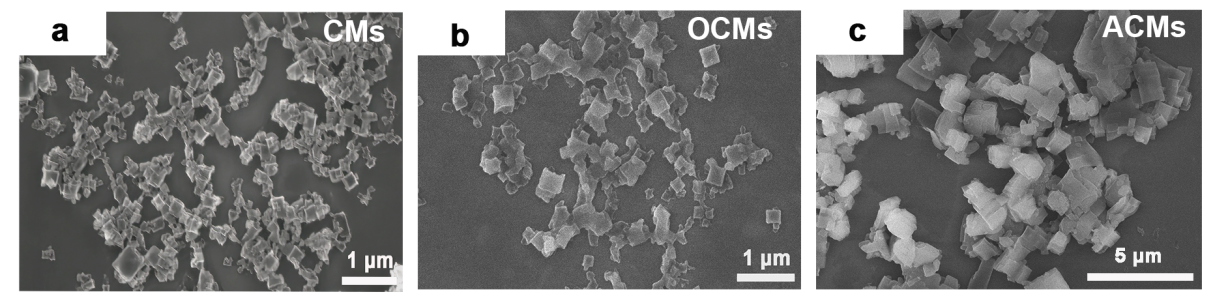


**Figure S2.** SEM images of (a) CMs, (b) OCMs, and (c) ACMs.


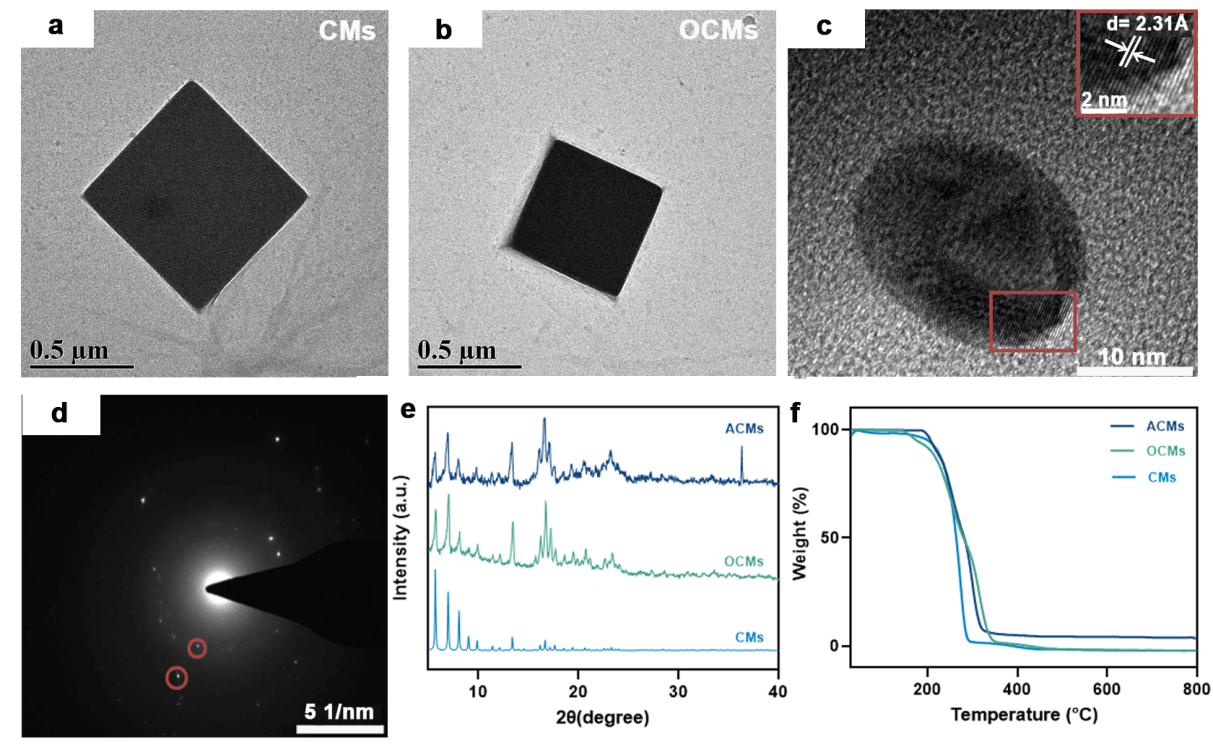


**Figure S3.** TEM images of (a) CMs and (b) OCMs. (c) Lattice and (d) electron diffraction of AgNPs. (e) XRD patterns of CMs, OCMs, and ACMs. (f) TGA curves of CMs, OCMs, and ACMs.


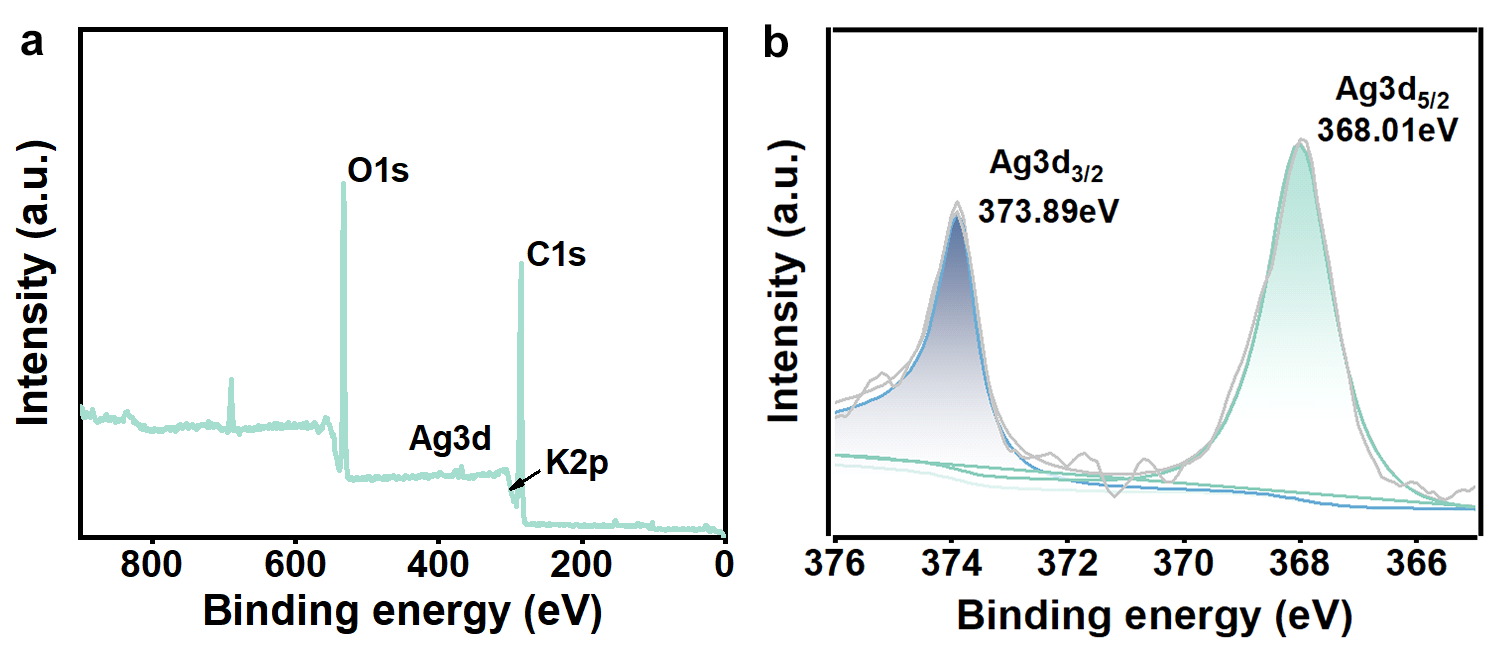


**Figure S4.** (a) High-resolution XPS spectra of ACMs, (b) high-resolution XPS spectra of Ag 3d of ACMs.


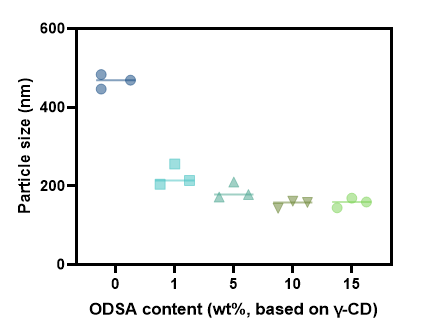


**Figure S5.** Particle size of the ACMs with different ODSA contents.


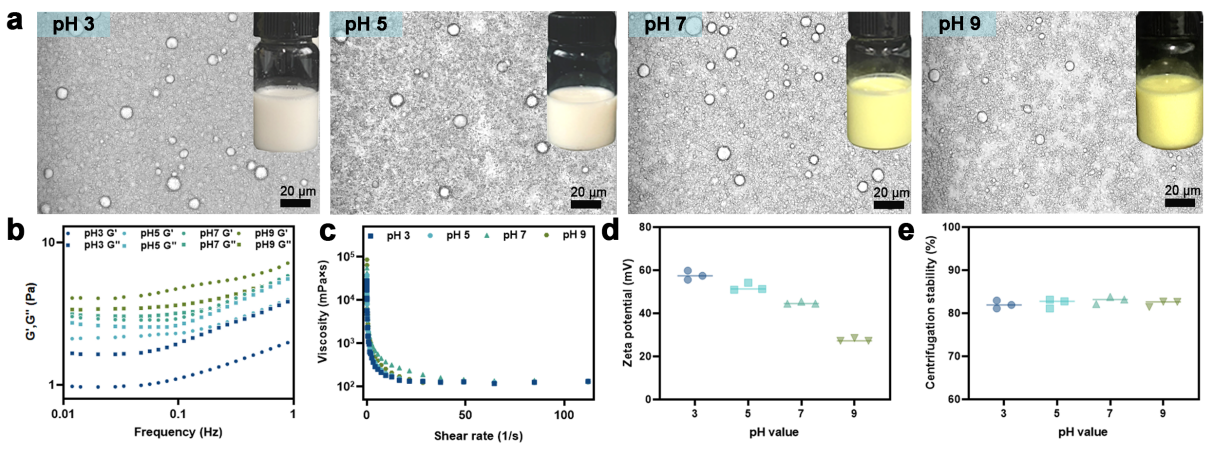


**Figure S6.** (a) Microscopic images, (b) dynamic scanning curves of storage modulus and loss modulus as a function of frequency, (c) viscosity, (d) potential, and (e) centrifugal stability constants of PE stabilized with ACMs at varying pHs.


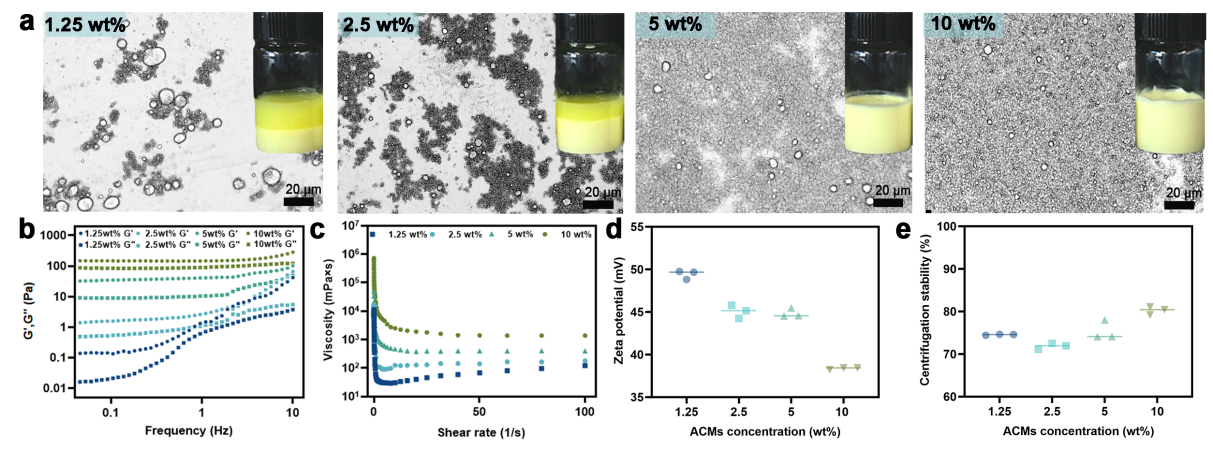


**Figure S7.** (a) Microscopic images, (b) dynamic scanning curves of storage modulus and loss modulus as a function of frequency, (c) viscosity, (d) potential, and (e) centrifugal stability constants of PE stabilized with ACMs at varying concentrations.


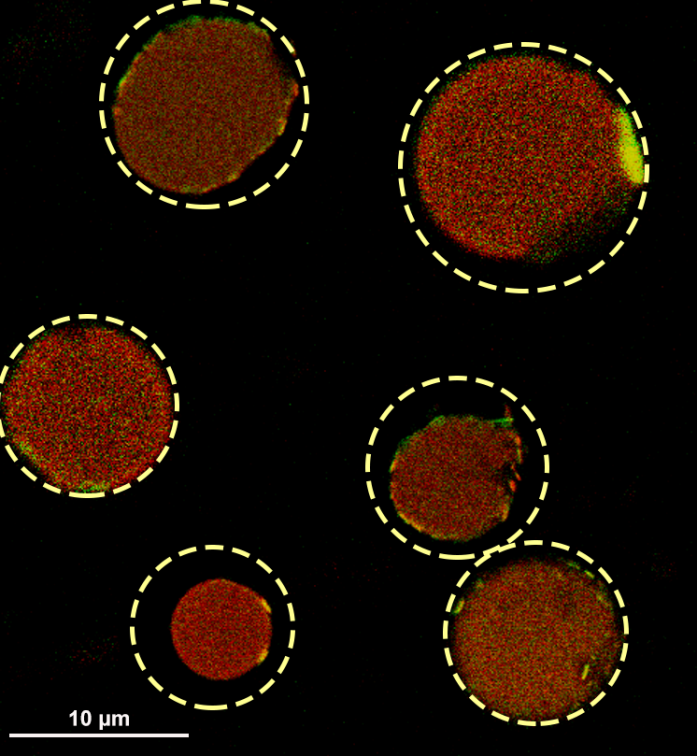


**Figure S8.** Confocal laser scanning microscopy of PE under optimal conditions.


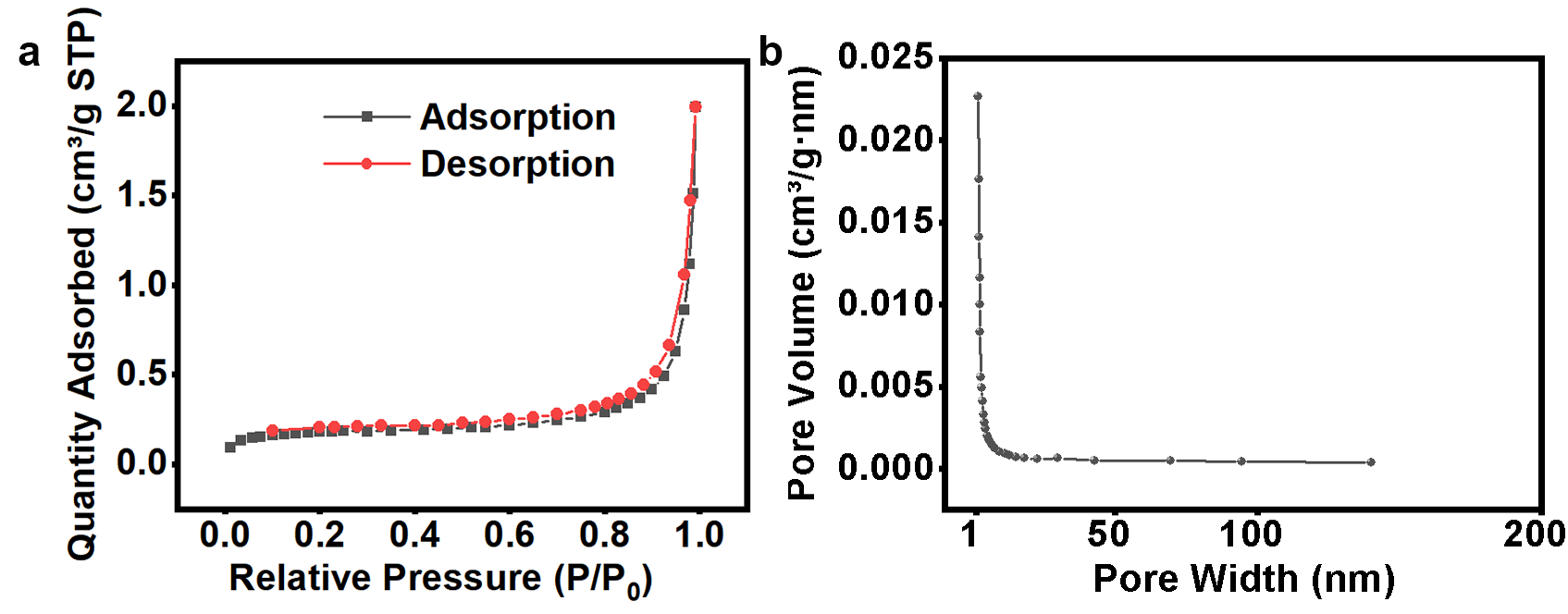


**Figure S9.** (a) Absorption and desorption curves of γ-CD. (b) Pore size curves of γ-CD.


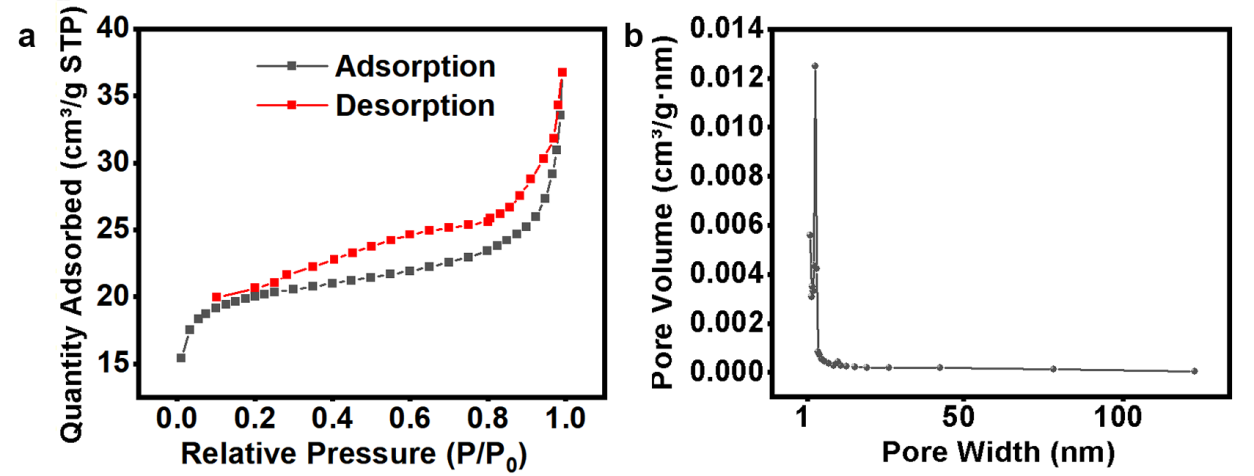


**Figure S10.** (a) Absorption and desorption curves of ACMs. (b) Pore size curves of ACMs.

**

**

**Figure S11.** Ethylene adsorption curves for ACMs and OCMs.


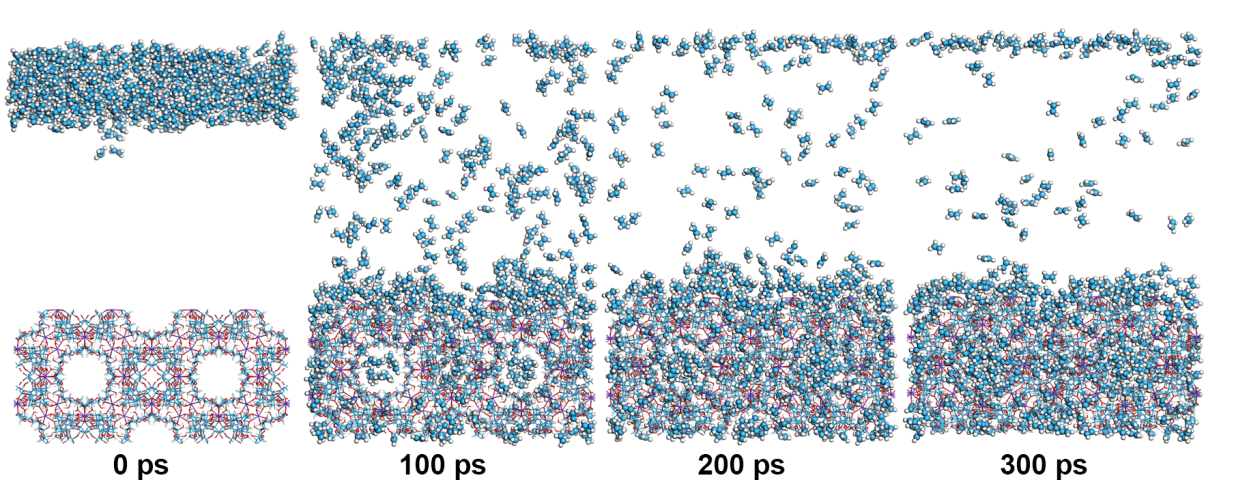


**Figure S12.** Adsorption of ethylene on CMs simulated by the Metropolis method over 300 ps.


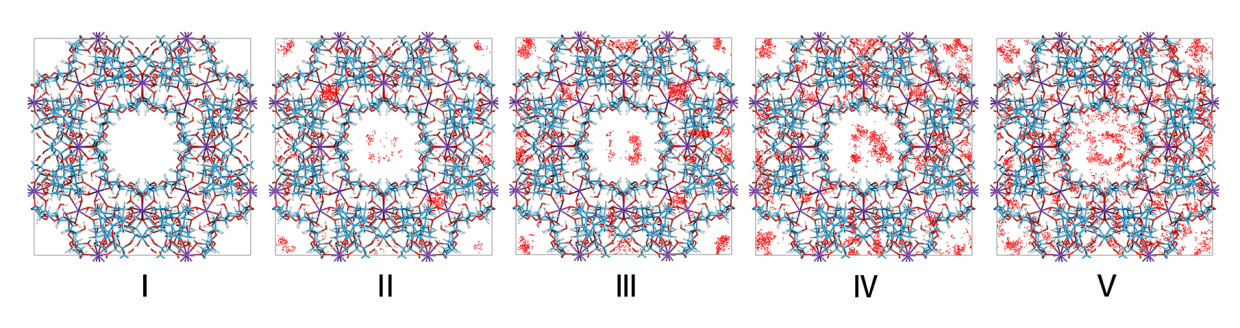


**Figure S13.** Ethylene distribution density within CMs at 298K, with densities increasing sequentially from I to V.


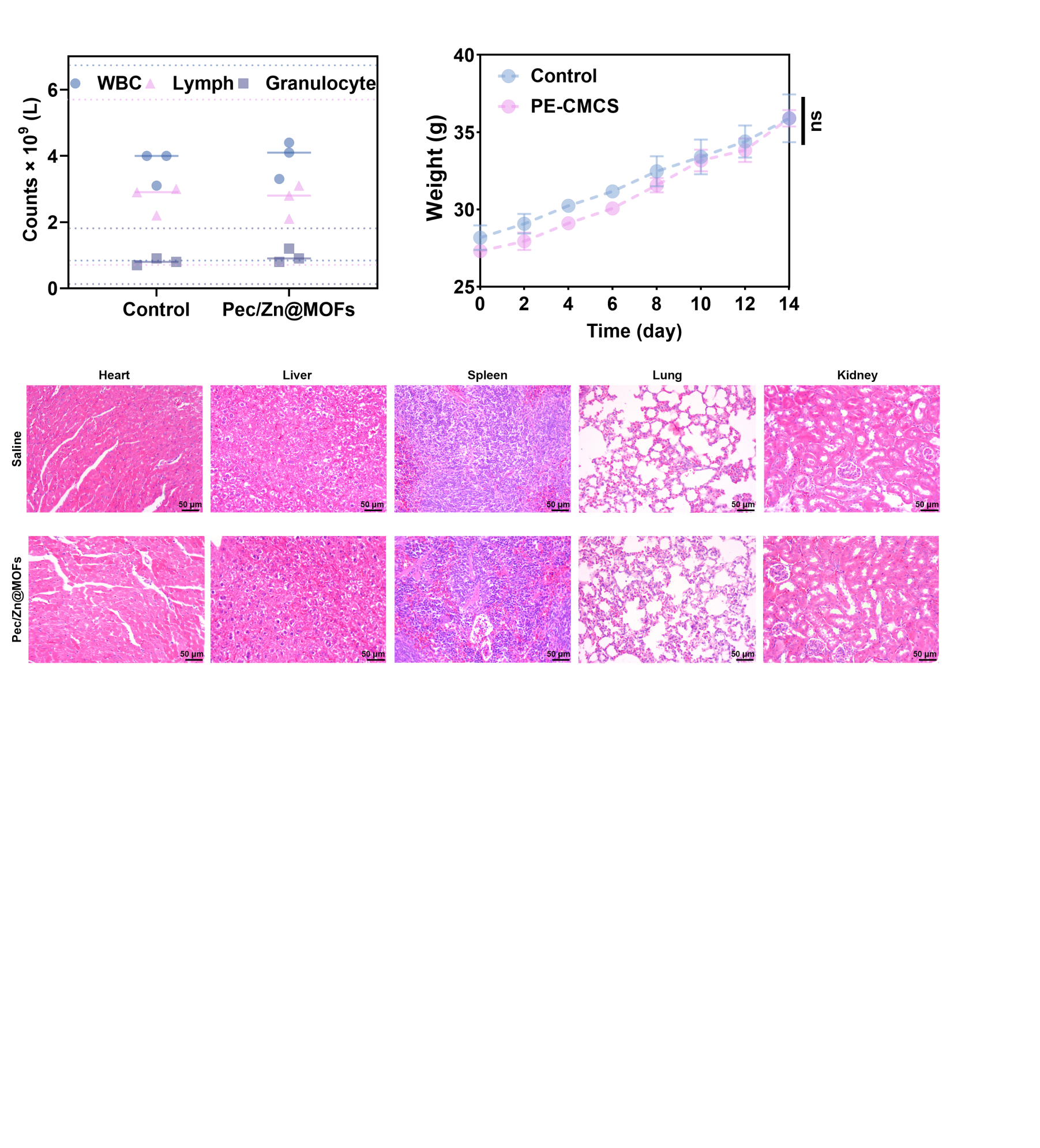


**Figure S14.** The body weight of mice was treated with saline (control) and PE-CMCS. Data are shown as mean ± SD (n = 6). *p* values were obtained by one-way ANOVA followed by Duncan’s post-hoc test. Statistical significance is set as **p* < 0.05, ***p* < 0.01, ****p* < 0.001, *****p* < 0.0001; ns, not statistically significant.


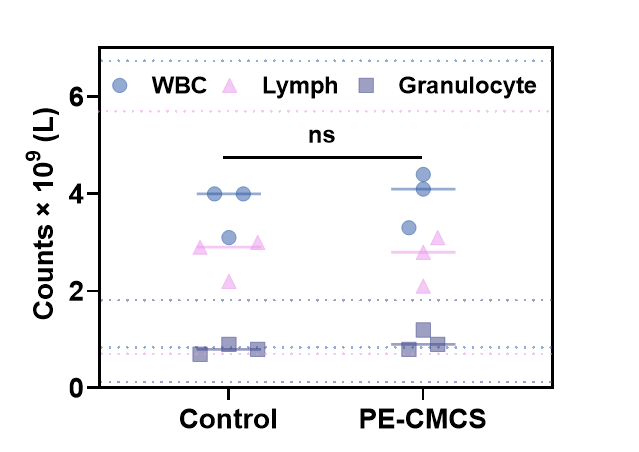


**Figure S15.** WBC, granulocyte, and lymph levels in the blood of rats in different treatment groups. Data are shown as mean ± SD (n = 3). *p* values were obtained by one-way ANOVA followed by Duncan’s post-hoc test. Statistical significance is set as **p* < 0.05, ***p* < 0.01, ****p* < 0.001, *****p* < 0.0001; ns, not statistically significant.

**
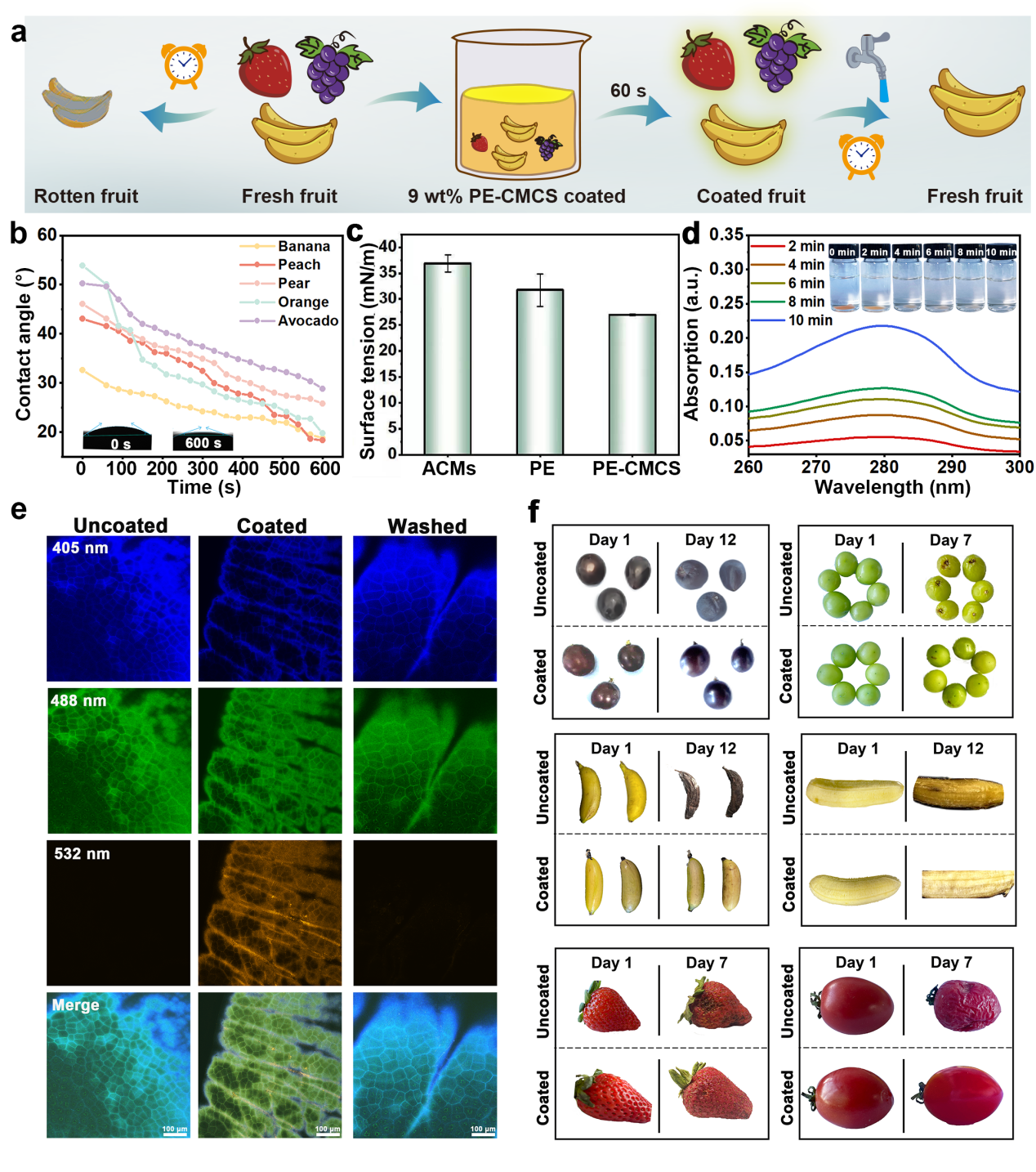
**

**Figure S16.** Schematic representation of the fruit coating and washing process.


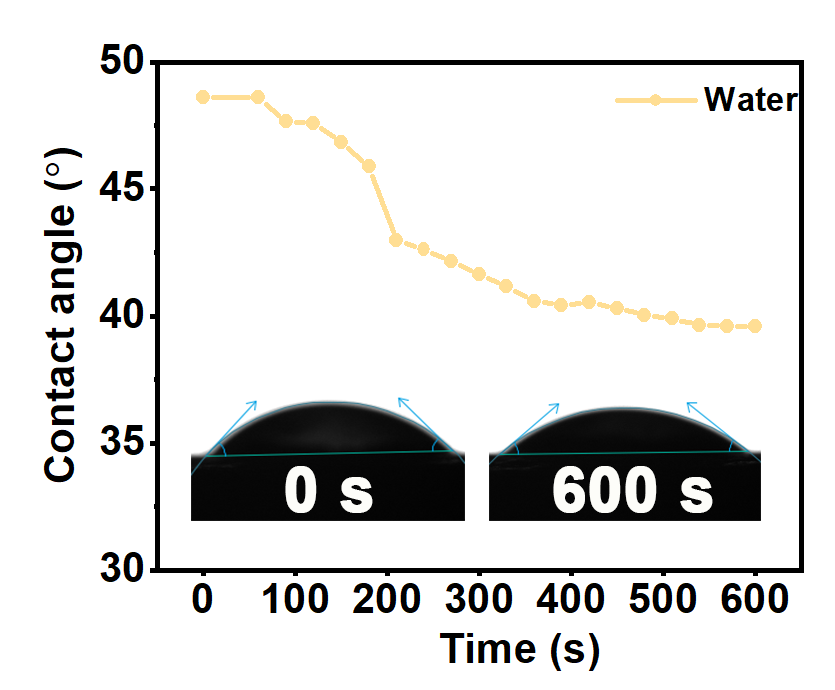


**Figure S17.** Contact angles of water on the banana surface at different times.


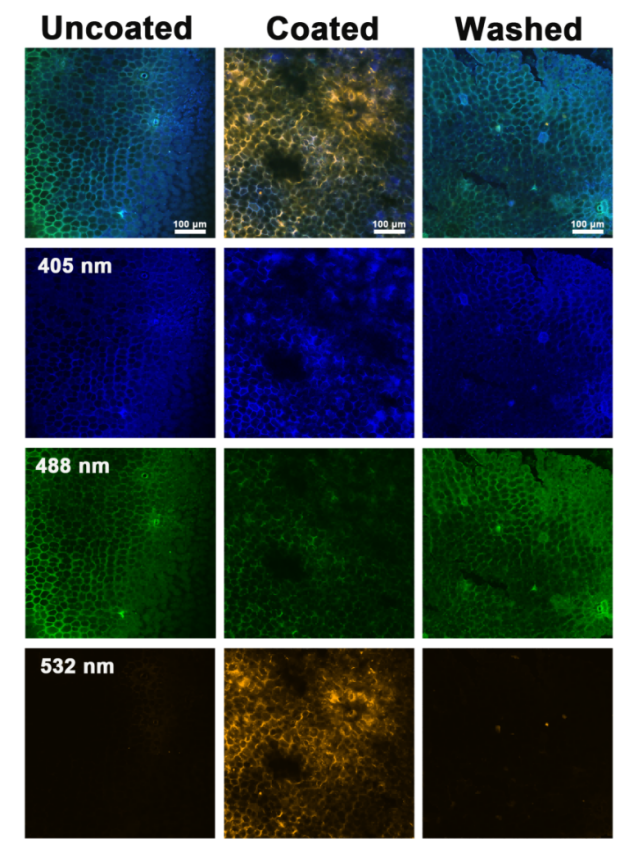


**Figure S18.** CLSM images of uncoated, coated, and post-washing coated emperor bananas at different excitation wavelengths.


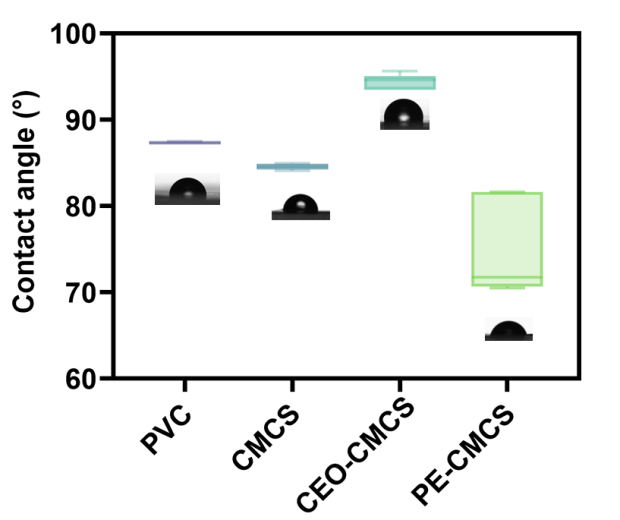


**Figure S19.** Water contact angle of PVC, CMCS, CEO-CMCS, and PE-CMCS films.

**
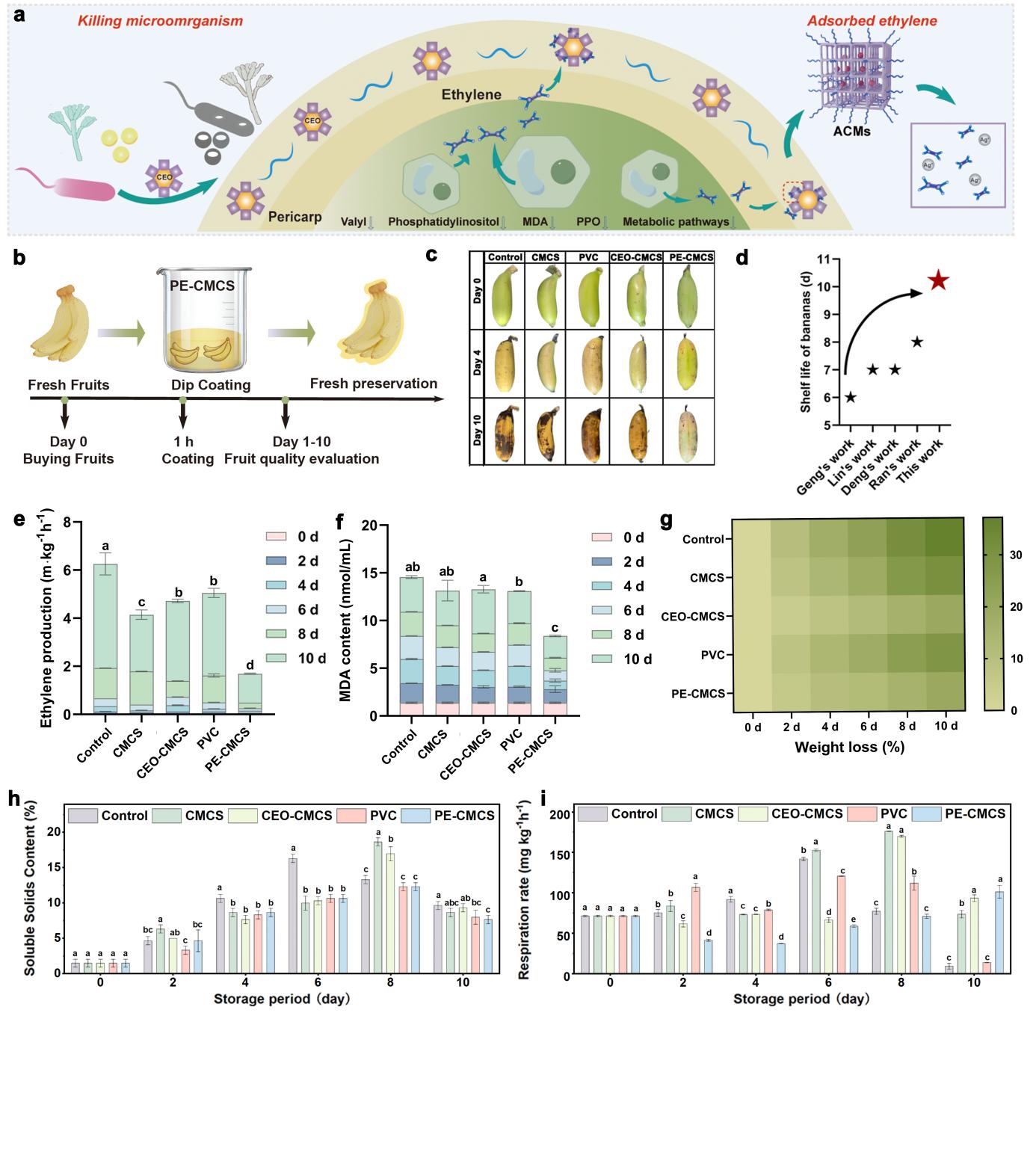
**

**Figure S20.** The preservation mechanism for emperor bananas using PE-CMCS.


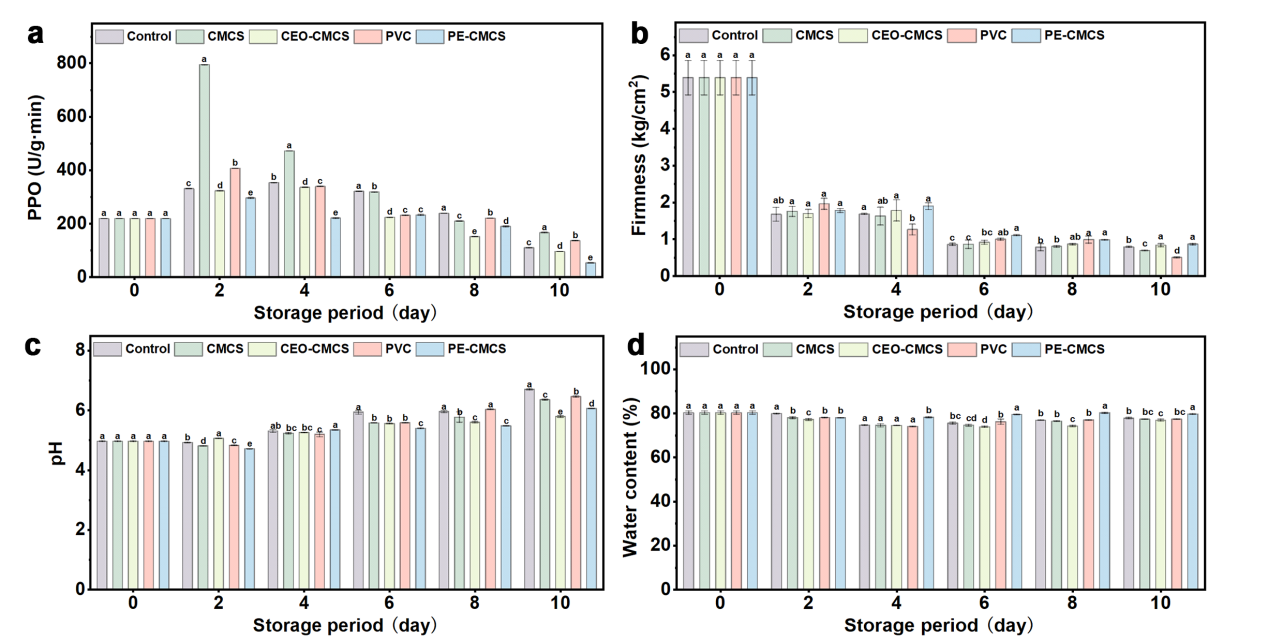


**Figure S21.** (a) Polyphenol oxidase (PPO), (b) firmness, (c) pH, and (d) water content of emperor bananas under different fresh-keeping coatings.





**Figure S22.** Determination of silver ion standard curve using flame atomic absorption spectroscopy.


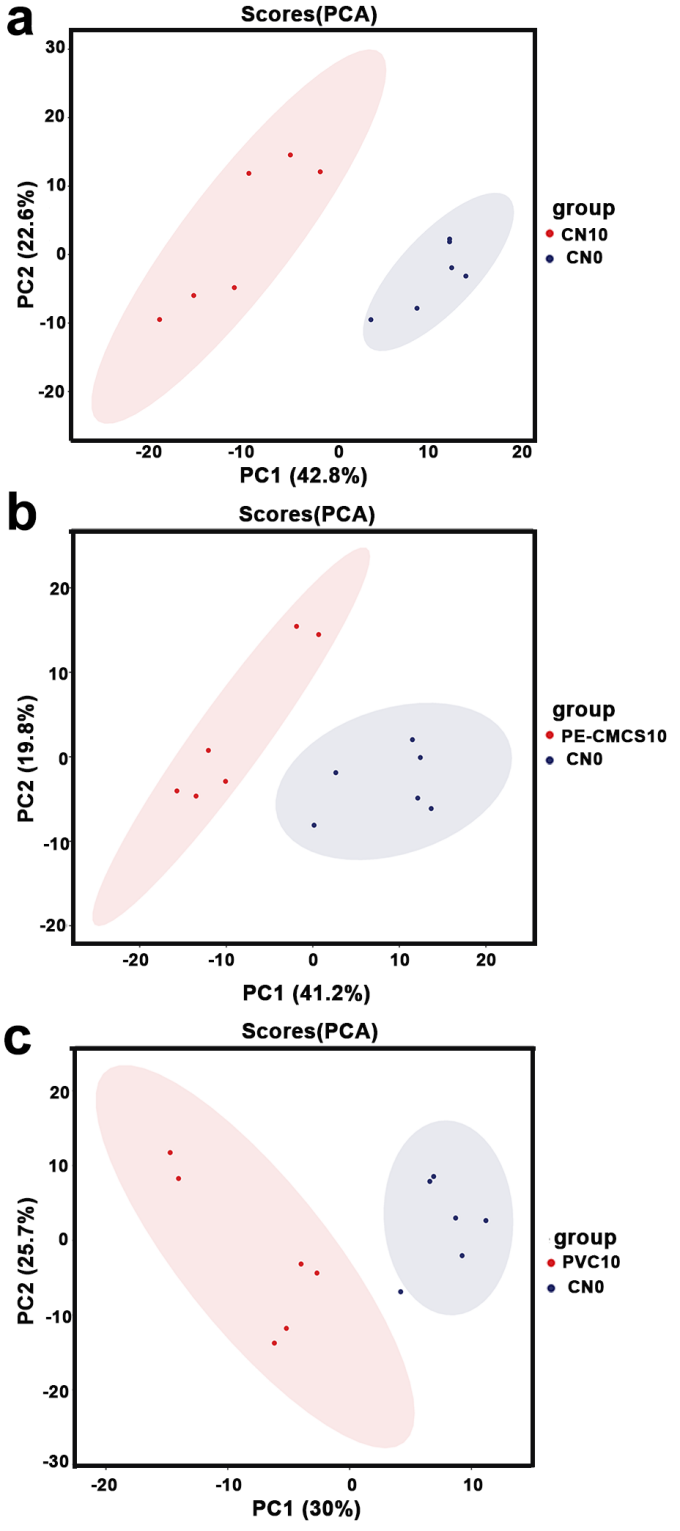


**Figure S23.** PCA score plots with (a) CN10 vs CN0, (b) PE-CMCS10 vs CN0, and (c) PVC10 vs CN0.


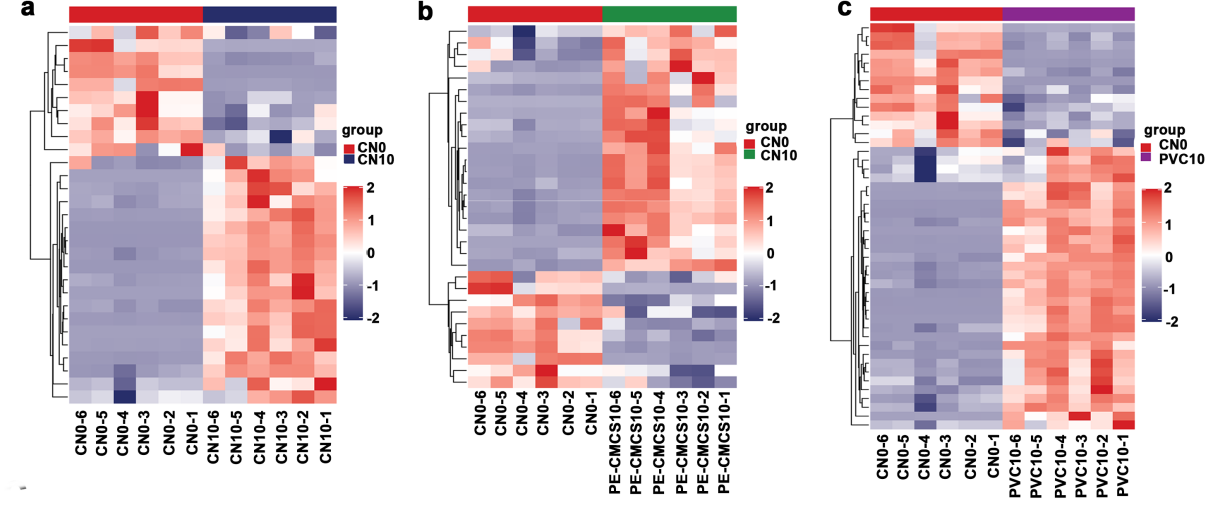


**Figure S24.** Clustering heatmap visualization of differential metabolites with (a) CN10 vs CN0, (b) PE-CMCS10 vs CN0, and (c) PVC10 vs CN0.


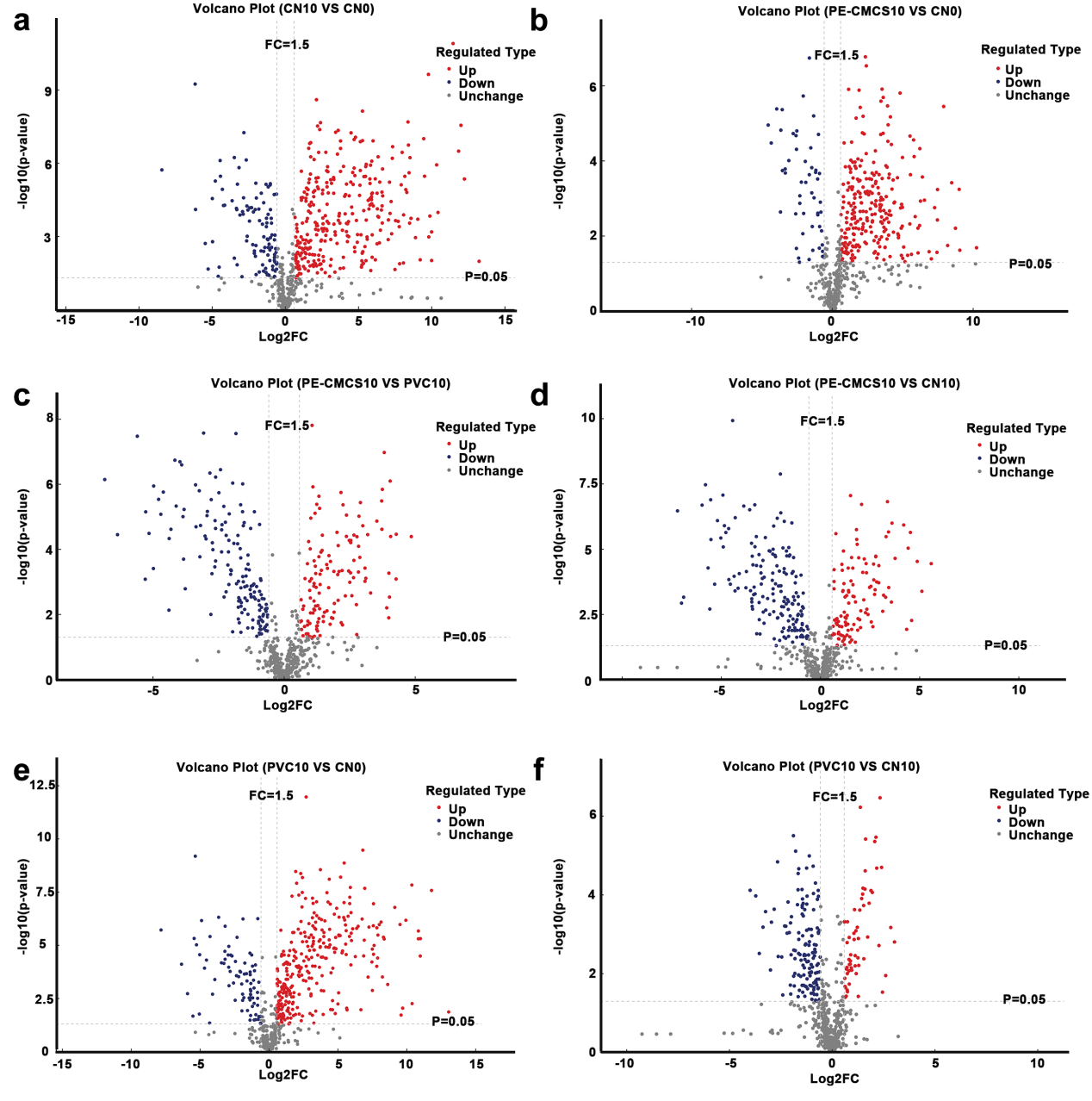


**Figure S25.** Volcano plot of the metabolites identified with (a) CN10 vs CN0, (b) PE-CMCS10 vs CN0, (c) PE-CMCS10 vs PVC10, (d) PE-CMCS10 vs CN10, (e) PVC10 vs CN0, and (f) PVC10 vs CN10.


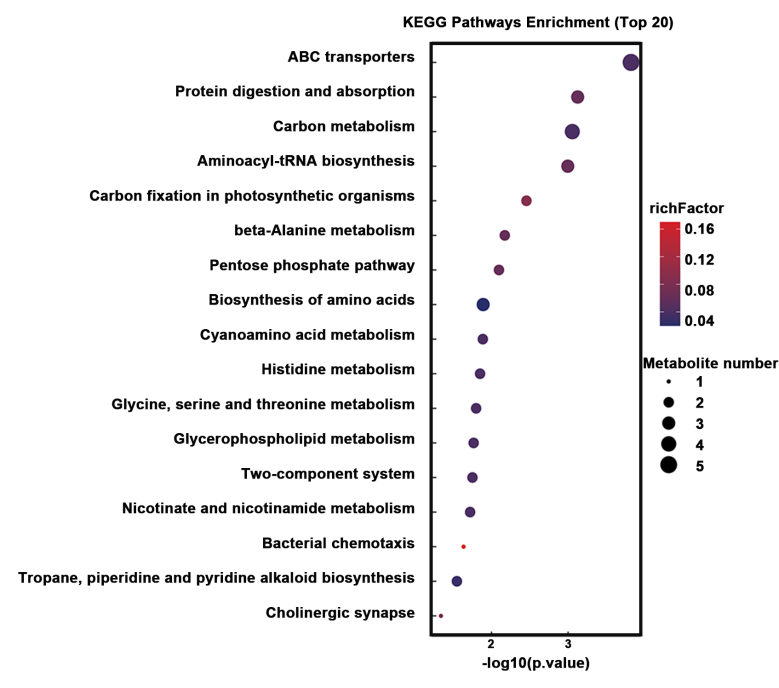


**Figure S26.** KEGG pathway enrichment analysis of differential metabolites in groups with CN10 vs CN0.


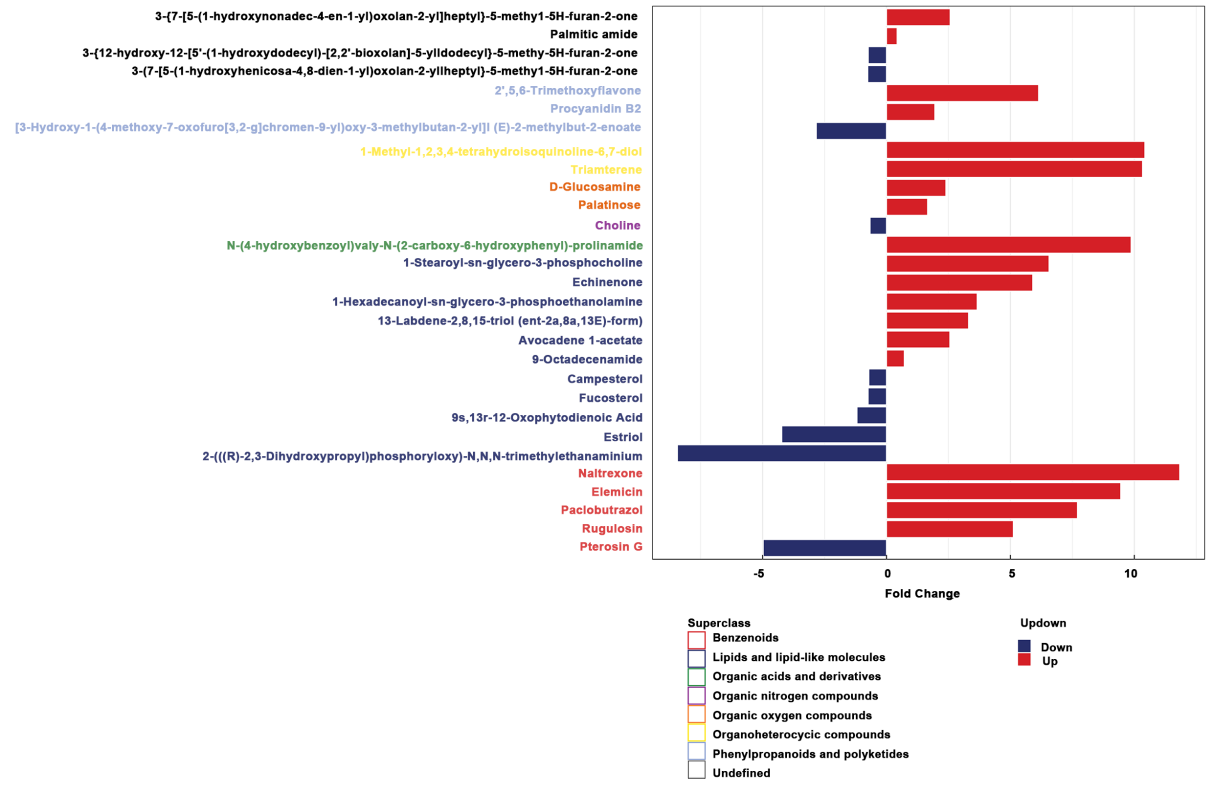


**Figure S27.** Significant difference in metabolite expression difference multiplicity analysis CN10 vs CN0.


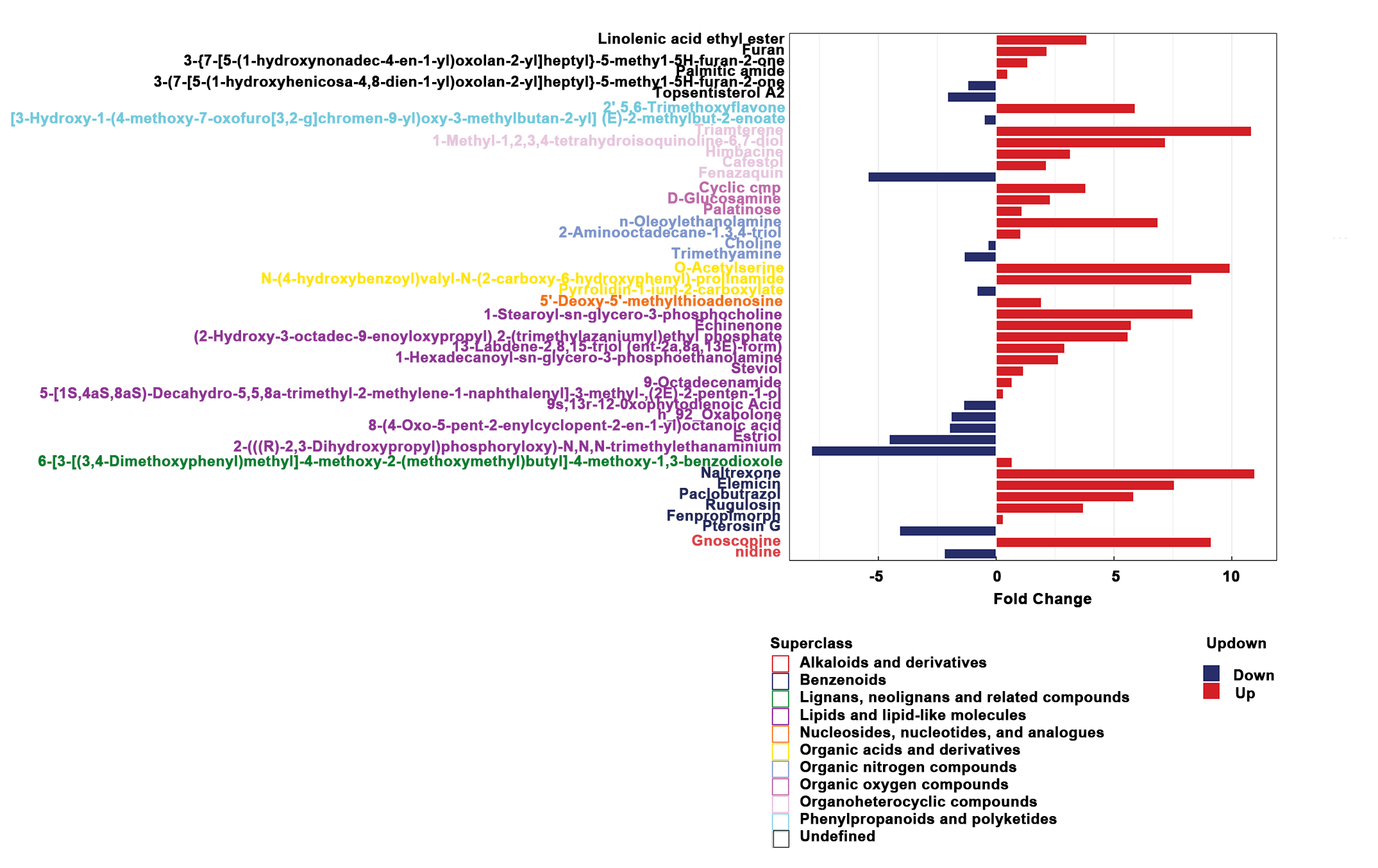


**Figure S28.** Significant difference in metabolite expression difference multiplicity analysis PVC10 vs CN0.


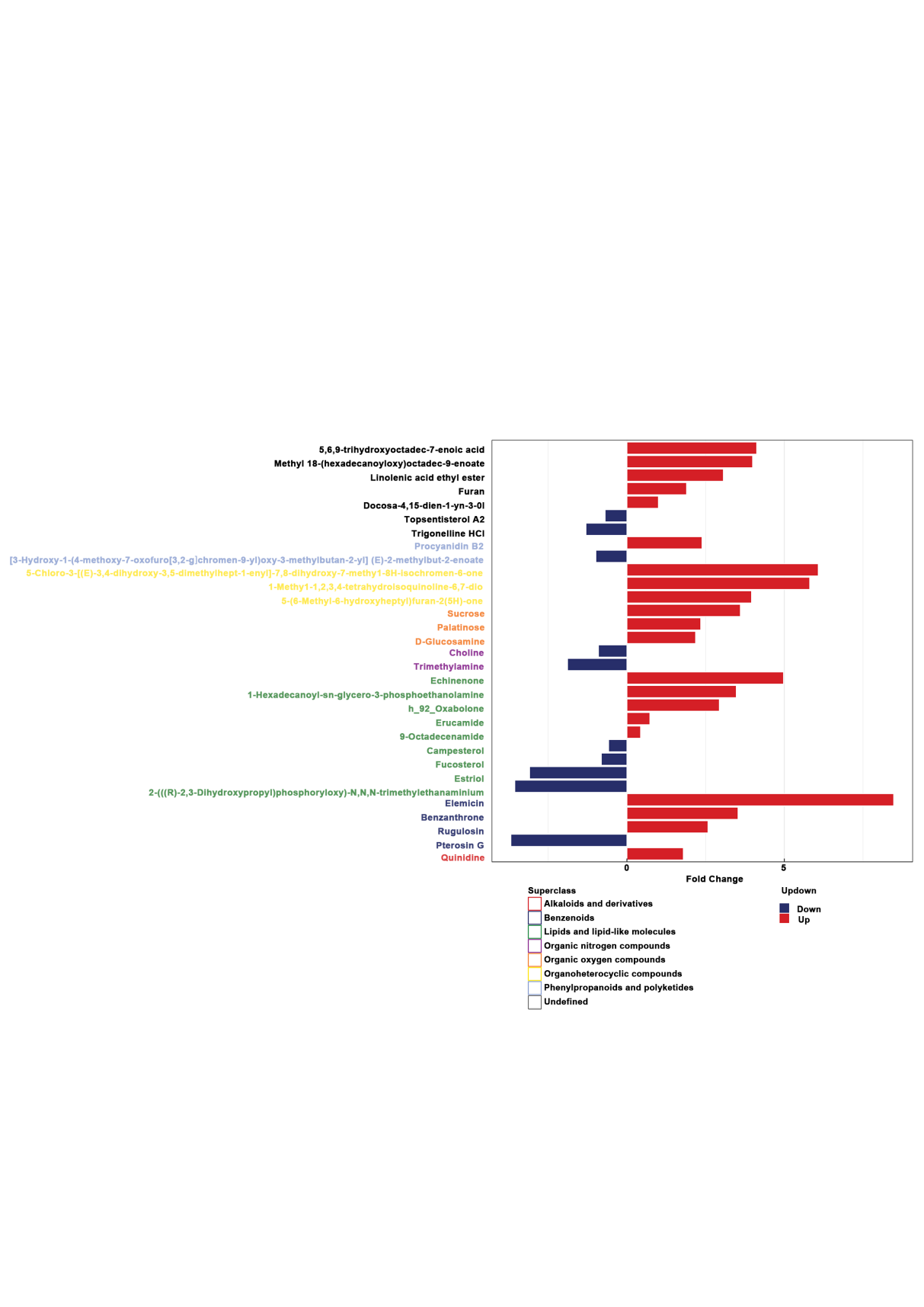


**Figure S29.** Significant difference in metabolite expression difference multiplicity analysis PE-CMCS10 vs CN0.

**3. Supplementary Tables**

**Table S1.** Colorimetric data of PVC, CMCS, CEO-CMCS, and PE-CMCS

| Sample | *L** | *a** | *b** | *c** | *h** |
| --- | --- | --- | --- | --- | --- |
| PVC | 91.10±0.16 | −1.58±0.045 | −3.78±0.084 | 4.10±0.10 | 246.74±0.58 |
| CMCS | 89.08±0.11 | −1.38±0.045 | −2.10±0.10 | 2.54±0.055 | 236.00±0.87 |
| CEO-CMCS | 88.26±0.17 | −2.70±0.071 | 6.46±0.97 | 7.00±0.91 | 111.22±2.6 |
| PE-CMCS | 86.50±0.48 | −1.32±0.11 | 4.78±1.0 | 4.98±0.94 | 106.08±4.1 |

**Table S2.** The average migration quantity of silver ions in emperor bananas with PE-CMCS

|  | 0 d | 4 d | 10 d |
| --- | --- | --- | --- |
| Peel | ND | ND | ND |
| Flesh | ND | ND | ND |

ND= not detected

**Table S3**. Input and output (life cycle inventory) of the PE-CMCS synthesis method presented in this work for the life cycle carbon and cost analysis.

| Input/Output | Materials | CAS | Corresponding database | Quantity | Unit | Cost/$ |
| --- | --- | --- | --- | --- | --- | --- |
| The synthesis of PE-CMCS | | | | | | |
| Input* |  |  |  |  |  |  |
| Energy |  |  | Electricity, high voltage {CN}\| market group for \| APOS, U | 0.0148 | Kwh | 0.00099 |
| Deionized water |  |  | Water, deionised {RoW}\| market for water, deionised \| APOS, U | 0.4 | L | 0.00016 |
| ACMs |  |  |  | 0.001 | Kg | 5.83 |
| CEO | Clove Essential Oil | 8000-34-8 | Clove essential oil{CN}\| market for clove essential oil \| APOS, U | 0.001 | L | 0.013 |
| CMCS | Carboxymethyl Chitosan | 83512-85-0 | Carboxymethyl chitosan{CN}\| market for carboxymethyl chitosan \| APOS, U | 0.016 | Kg | 0.47 |
| Output* |  |  |  |  |  |  |
| PE-CMCS |  |  |  | 0.42（Suitable for 10 Kg of emperor bananas） | L | 6.31 |

The input and output data of ACMs (1g) are as follows:

| Input/Output | Materials | CAS | Corresponding database | Quantity | Unit | Cost/$ |
| --- | --- | --- | --- | --- | --- | --- |
| The synthesis of Ag@CD-MOFs (ACMs) | | | | | | |
| Input* |  |  |  |  |  |  |
| Energy |  |  | Electricity, high voltage {CN}\| market group for \| APOS, U | 68.62 | Kwh | 4.6 |
| γ-CD | γ-Cyclodextrin | 17465-86-0 | Cyclodextrin {GLO}\| market for cyclodextrin \| APOS, U | 0.0042 | Kg | 0.2595 |
| ODSA | Octadecenylsuccinic Anhydride | 28777-98-2 | Octadecenylsuccinic anhydride {GLO}\| market for octadecenylsuccinic anhydride \| APOS, U | 0.000042 | Kg | 0.00018 |
| AgNO_3_ | Silver Nitrate | 7761-88-8 | Silver nitrate {CN}\| market for silver nitrate \| APOS, U | 4.25 × 10^−8^ | g | 5.7 × 10^−9^ |
| Acetonitrile | Acetonitrile | 75-05-8 | Acetonitrile {CN}\| market for acetonitrile \| APOS, U | 0.0075 | L | 0.67 |
| KOH | Potassium Hydroxide | 1310-58-3 | Potassium hydroxide {CN}\| market for potassium hydroxide \| APOS, U | 0.00096 | Kg | 0.00081 |
| Isopropanol | Isopropanol | 67-63-0 | Isopropanol {GLO}\| market for isopropanol \| APOS, U | 0.365 | L | 0.26 |
| CTAB | Cetyl Trimethyl Ammonium Bromide | 57-09-0 | Cetyl trimethyl ammonium bromide {ROW}\| market for Cetyl trimethyl ammonium bromide \| APOS, U | 0.00072 | Kg | 0.0025 |
| Methyl Alcohol | Methyl Alcohol | 67-56-1 | Methyl alcohol {CN}\| market for methyl alcohol \| APOS, U | 0.06 | L | 0.019 |
| Hexane | Hexane | 110-54-3 | Hexane {CN}\| market for methyl hexanel \| APOS, U | 0.015 | L | 0.013 |
| Deionized water |  |  | Water, deionised {RoW}\| market for water, deionised \| APOS, U | 0.13 | L | 0.000052 |
| Output* |  |  |  |  |  |  |
| ACMs |  |  |  | 0.001 | Kg | 5.83 |

**4. Supplementary References**

[1] B. Sun, D. Wang, Y. Jiang, R. Wang, L. Lyu, G. Diao, W. Zhang, H. Pang, “Cyclodextrin Metal–Organic Framework Functionalized Carbon Materials with Optimized Interface Electronics and Selective Supramolecular Channels for High-Performance Lithium–Sulfur Batteries,” *Advanced Materials* (2024**)**: 2415633, <https://doi.org/10.1002/adma.202415633>

[2] H. Alotaibi, E. Chung, S. H. Chung, G. Ren, V. Singh, J. Huang, “Sustainable γ-Cyclodextrin Frameworks Containing Ultra-Fine Silver Nanoparticles with Enhanced Antimicrobial Efficacy,” *Carbohydrate Polymers* (2023**)**: 120516, <https://doi.org/10.1016/j.carbpol.2022.120516>

[3] X. Yu, X. Li, S. Ma, Y. Wang, W. Zhu, H. Wang, “Biomass-Based, Interface Tunable, and Dual-Responsive Pickering Emulsions for Smart Release of Pesticides,” *Advanced Functional Materials* (2023**)**: 2214911, <https://doi.org/10.1002/adfm.202214911>

[4] Y. Cui, Y. Cheng, Z. Xu, B. Li, W. Tian, J. Zhang, “Cellulose-Based Transparent Edible Antibacterial Oxygen-Barrier Coating for Long-Term Fruit Preservation,” *Advanced Science* (2024**)**: 2409560, <https://doi.org/10.1002/advs.202409560>

[5] Y.-P. Li, Y.-N. Zhao, S.-N. Li, D.-Q. Yuan, Y.-C. Jiang, X. Bu, M.-C. Hu, Q.-G. Zhai, “Ultrahigh-Uptake Capacity-Enabled Gas Separation and Fruit Preservation by a New Single-Walled Nickel–Organic Framework,” *Advanced Science* (2021**)**: 2003141, <https://doi.org/10.1002/advs.202003141>

[6] Y. Wang, Y. Lang, Q. Yang, P. Wu, “Breaking the Photostability and pH Limitation of Halo-Fluoresceins through Chitosan Conjugation,” *Advanced Materials* (2023**)**: 2210956, <https://doi.org/10.1002/adma.202210956>

[7] Z. Zhu, C. Li, Y. Lin, L. Li, K. Liu, W. Wen, S. Ding, C. Zhou, Y. Lai, B. Luo, “Versatile 3D Printing Scaffold with Spatiotemporal Release of Multiple Drugs for Bone Regeneration,” *ACS Nano* (2025**)**: 13637, <https://doi.org/10.1021/acsnano.4c13265>

[8] X. Wu, Q. Borjihan, Y. Su, H. Bai, X. Hu, X. Wang, J. Kang, A. Dong, Y.-W. Yang, “Supramolecular Switching-Enabled Quorum Sensing Trap for Pathogen-Specific Recognition and Eradication to Treat Enteritis,” *Journal of the American Chemical Society* (2024**)**: 35402, <https://doi.org/10.1021/jacs.4c14424>

[9] S. Jung, Y. Cui, M. Barnes, C. Satam, S. Zhang, R. A. Chowdhury, A. Adumbumkulath, O. Sahin, C. Miller, S. M. Sajadi, L. M. Sassi, Y. Ji, M. R. Bennett, M. Yu, J. Friguglietti, F. A. Merchant, R. Verduzco, S. Roy, R. Vajtai, J. C. Meredith, J. P. Youngblood, N. Koratkar, M. M. Rahman, P. M. Ajayan, “Multifunctional Bio-Nanocomposite Coatings for Perishable Fruits,” *Advanced Materials* (2020**)**: 1908291, <https://doi.org/10.1002/adma.201908291>

[10] Y. Ning, W. Yang, S. Liu, J. Xu, X. Cheng, S. Xu, J. Li, L. Wang, “A Fluorescent Polyvinyl Alcohol Film with Efficient Photodynamic Antimicrobial Performance Enabled by Berberine/Phytic Acid Salt for Food Preservation,” *Advanced Functional Materials* (2025**)**: 2411314, <https://doi.org/10.1002/adfm.202411314>

[11] G. Chen, K. Wang, P. Chen, D. Cai, Y. Shao, R. Xia, C. Li, H. Wang, F. Ren, X. Cheng, Y. Yu, “Fully Biodegradable Packaging Films for Fresh Food Storage Based on Oil-Infused Bacterial Cellulose,” *Advanced Science* (2024**)**: 2400826, <https://doi.org/10.1002/advs.202400826>

[12] F. Liu, L. Kuai, C. Lin, M. Chen, X. Chen, F. Zhong, T. Wang, “Respiration-Triggered Release of Cinnamaldehyde from a Biomolecular Schiff Base Composite for Preservation of Perishable Food,” *Advanced Science* (2024**)**: 2306056, <https://doi.org/10.1002/advs.202306056>
